# Meprin β Modulates Brevican Proteolysis Impairing Neural Plasticity and Memory Formation

**DOI:** 10.1096/fj.202500017R

**Published:** 2025-05-21

**Authors:** Maximilian Keller, Celine Gallagher, Simon Kreiselmaier, Kira Bickenbach, Ulrich Schmitt, Liana Marengo, Dayan Taghikhah, Mohammad Abukhalaf, Andreas Tholey, Christoph Becker‐Pauly, Thomas Mittmann, Claus U. Pietrzik

**Affiliations:** ^1^ Institute for Pathobiochemistry University Medical Center Mainz Germany; ^2^ Institute for Physiology University Medical Center Mainz Germany; ^3^ Unit for Degradomics of the Protease Web, Institute of Biochemistry Christian‐Albrechts‐University Kiel Kiel Germany; ^4^ Leibniz‐Institute for Resilience Research Mainz Germany; ^5^ Systematic Proteome Research & Bioanalytics, Institute for Experimental Medicine Christian‐Albrechts‐Universität Zu Kiel Kiel Germany; ^6^ Molecular Neurodegeneration, Institute for Pathobiochemistry University Medical Center Mainz Mainz Germany

**Keywords:** brevican, extracellular matrix, long‐term potentiation, memory consolidation, meprin β

## Abstract

The metalloprotease meprin β is known for its multifunctional involvement in various physiological processes throughout the body including the brain. However, its broader functions within the brain besides amyloid β generation remain largely unexplored. To investigate this, we utilized a mouse model overexpressing meprin β in neurons within the cortex and hippocampus, regions crucial for learning and memory. Behavioral assessments, employing the Morris' Water Maze paradigm test, revealed impaired cognitive functions in animals overexpressing meprin β. Furthermore, electrophysiological recordings in hippocampal slices using multielectrode arrays showed an impaired long‐term potentiation (LTP) in meprin β‐overexpressing mice compared to wild‐type counterparts. Intriguingly, concomitant with the LTP impairment, we observed an increased neuronal excitability. These findings underline the complicated interplay between meprin β abundance and behavioral manifestations, suggesting a broader impact on neural circuit dynamics. To elucidate the molecular mechanisms underlying these observed deficits, western blotting analyses were conducted to address the expression of glutamatergic receptors. Neither the expression of the N‐methyl‐D‐aspartate (NMDA) nor the α‐amino‐3‐hydroxy‐5‐methyl‐4‐isoxazolepropionic acid (AMPA) receptor showed variation relative to each other. The application of N‐terminomics identified brevican as a proteolytic substrate of meprin β and thus a potential key mediator linking meprin β overexpression to the observed effects. Previous studies have reported that brevican knockout in animal models influences learning and memory. Our data demonstrate that meprin β modulates brevican expression, likely contributing to the effects we have observed in our mouse model. These results shed light on the broader functional significance of meprin β in neurological processes.

## Graphical abstract

Meprin β was studied using mice overexpressing it in cortical and hippocampal neurons. N‐terminomics identified brevican, a key extracellular matrix protein associated with synaptic plasticity and memory, as a potential substrate of meprin β. Further analyses revealed that brevican levels are dependent on the catalytic activity of meprin β. These findings provide an explanation for the effects observed in the behavioral test and electrophysiological analyses, highlighting meprin β's broader neurological role in cognitive function.

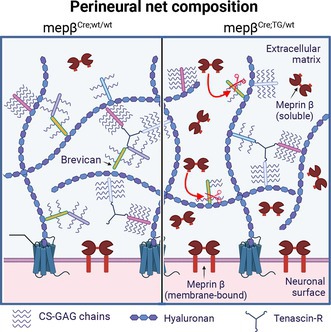

**Abbreviations**
ADAMTS4: A Disintegrin and Metalloproteinase with Thrombospondin Motifs
AMPA: α‐amino‐3‐hydroxy‐5‐methyl‐4‐isoxazolepropionic acid
APP: amyloid precursor protein
Cx: cortex
ECM: extracellular matrix
fEPSP: field excitatory postsynaptic potentials
HC: hippocampus
HFS: high‐frequency stimulus
HUNTER: High‐efficiency Undecanal‐based N Termini EnRichment
LTP: long‐term potentiation
MEA: microelectrode array
mepβ^Cre;TG/wt^
: meprin β—overexpressing
mepβ^Cre;wt/wt^
: wild‐type
MMP: matrix metalloprotease
NMDA: N‐methyl‐D‐aspartate
PNN: perineural nets
PPR: paired‐pulse ratio
PSD‐95: postsynaptic density density‐95

## 1 Introduction

Although meprin β was first identified over 40 years ago, the complete range of its biological functions remains unclear. Meprin β is a membrane‐bound, zinc‐dependent metalloprotease that is a member of the astacin family of proteases [1]. The enzyme is anchored to the cell surface via a C‐terminal transmembrane domain, positioning it to interact with extracellular surface proteins [2]. Pro‐meprin β is cleaved and activated by Matriptase‐2 or other tryptic proteases [3]. Additionally, meprin β itself can be shed from the membrane and exert its functional role in a soluble form, where it is primarily involved in the cleavage of extracellular matrix (ECM) proteins, particularly procollagen and proteoglycans [4]. Moreover, Bond and Beynon [5] reported that cleavage of meprin β is crucial for tissue remodeling and regulating inflammatory responses, akin to other members of the astacin family. Key substrates of meprin β comprise E‐cadherin, procollagen, galectin‐3, and Mucin 2 [6, 7, 8, 9, 10]. In particular, the cleavage of Mucin 2 in the small intestine plays a key role in mucosal defense by aiding in the removal of bacteria through the breakdown of mucin networks. Additionally, meprin β is highly expressed in the brush‐border membranes of proximal tubule epithelial cells of kidneys, where it is involved in the regulation of ECM homeostasis. Dysregulation of its activity has been implicated in pathological conditions, including fibrosis and chronic kidney disease [11].

Recent studies have highlighted a role for meprin β in the central nervous system, where meprin β is expressed in various cell types including neurons, suggesting the likelihood of an additional function beyond its established roles in the intestine and kidney. We and others have demonstrated the role of meprin β in the cleavage of the amyloid precursor protein (APP), a mechanism linked to the generation of amyloid‐β peptides, which are essential to the pathogenesis of Alzheimer's disease [11, 12]. This suggests that meprin β is an enzyme that exerts its function in the brain, which in turn could affect neurophysiological processes. Ligt et al. [13] showed that meprin β is associated with severe cognitive diseases. Its involvement in cognitive processes is further supported by uncovering that meprin β knockout mice have shown improvements in learning and memory compared to wild‐type controls, as demonstrated by enhanced performance in the Morris Water Maze paradigm test [14]. These findings may point to a potential role in brain ECM remodeling and therefore involvement in cognitive processes.

The ECM of the brain is different from that of other tissues. While fibronectins and collagens are abundant in the ECM of most tissues, the brain's ECM primarily consists of proteoglycans, tenascins, and hyaluronic acid [15]. Among the proteoglycans, brevican stands out as the shortest member of the aggrecan‐versican family. This group of proteoglycans is commonly referred to as the Lectican family, due to its structural similarities [16].

Brevican is proteolytically processed by “A Disintegrin and Metalloproteinase with Thrombospondin Motifs” (ADAMTS4), resulting in an N‐terminal 50 kDa fragment and a C‐terminal 90 kDa fragment in vivo. In addition to ADAMTS4, Brevican is also cleaved by several matrix metalloproteinases (MMPs), including MMP‐1, 2, 3, 7, 10, and 13 [17]. Furthermore, Armbrust et al. [18] showed that Brevican is a potential novel substrate in meprin β‐overexpressing astrocytes, highlighting the proteolytic involvement of meprin β in neural ECM remodeling.

To investigate the potential effect of meprin β on brain ECM, and therefore learning and memory, we utilized a mouse model overexpressing meprin β in the neurons of the cortex and hippocampus, regions crucial for memory consolidation. Assessment of novel meprin β substrates in the brain by performing High‐efficiency Undecanal‐based N Termini EnRichment (HUNTER) proteomics analysis and western analyses revealed brevican as a proteolytic ECM substrate in the brain. Additionally, behavioral paradigm tests and electrophysiological data corroborate a crucial interaction of brevican and meprin β in the hippocampus, emphasizing its potential role in ECM remodulation and therefore in learning and memory.

## 2 Materials and Methods

### 2.1 Animals

Meprin β‐overexpressing (mepβ^Cre;TG/wt^) and wild‐type (mepβ^Cre;wt/wt^) mouse strains were maintained on a 12 h light/dark cycle with food and water ad libitum.

*NEX* ^*Cre;TG/wt*^ *× Rosa26* ^*Mep1b‐HA*^ (mepβ^Cre;TG/wt^ or mepβ^Cre;wt/wt^) mice were generated by crossing *Rosa26* ^*Mep1b‐HA*^ mice [19] with *NEX* ^*Cre;TG/wt*^ mice [20]. We used 37 animals in total for protein expression, microelectrode array (MEA)‐, N‐terminomics, and behavioral experiments with mixed gender, since we did not expect any differences in phenotype. The animals have a C57BL/6 background, and breedings were genetically refreshed every 4–6 generations with wild‐type C57BL/6 animals. All animal studies were conducted in compliance with European and German guidelines for the care and use of laboratory animals and were approved by the Central Animal Facility of the University of Mainz and the ethical committee on animal care and use of Rhineland–Palatinate, Germany.

### 2.2 Synaptosome Isolation

Animals (*n* = 9) were sacrificed by cervical dislocation, and brains were removed. For further characterization, brains were dissected into cortex and hippocampus. To obtain enough protein, three hippocampi from each group were pooled. Functional synaptosomes were isolated as recommended by the manufacturer (ThermoFisher, 87793). Briefly, brain regions were weighed and mechanically homogenized in an appropriate amount of Syn‐PER reagent. First, the homogenate was centrifuged at 1200 *g* for 10 min. The supernatant was transferred to a fresh tube. After centrifugation at 15 000 *g* for 20 min, the pellet was resuspended in Syn‐PER reagent to a final concentration of 4–5 μg/μL, resulting in the synaptosome fraction. The synaptosome fraction was aliquoted and snap frozen for further analysis. The homogenate after the first centrifugation step and an aliquot of the supernatant after the last centrifugation step were snap frozen as well, resulting in the homogenate and S2 (cytosolic) fraction, respectively. The Syn‐PER reagent was supplemented with protease inhibitor cocktails prior to use (cOmplete, Roche).

### 2.3 Generation of Meprin β‐Deficient HEK293T Cell Line Using CRISPR/Cas9 Genome Editing

HEK293T cells were cultivated in DMEM GlutaMAX medium supplemented with 10% FCS. For CRISPR/Cas9 genome editing, multi‐guide RNA (G*U*C*UUCAGUAGGAAAUAGGC, C*A*A*GAGUUCCUCCACGCUCU, and U*U*A*UCCUGACAUAGUCAUCC) and recombinant *Streptococcus pyogenes* Cas9 2NLS (Synthego, Redwood City, CA, USA) were used to induce a deletion in MEP1B. Cas9 RNPs were prepared according to Synthego's instructions using 100 pmol multiguide RNA and 20 pmol Cas9 per electroporation using 7.6 × 10^4^ cells and the Neon device (ThermoFisher Scientific) with the following settings: 2 pulses, 1150 V, 20 ms. Immediately after electroporation, single‐cell seeding in 96‐well plates was performed to obtain single‐cell clones. From these, genomic DNA was extracted using the GeneJET Genomic DNA Purification Kit (Thermo Fisher Scientific) and genotyping PCRs with the following primers were performed: for: 5′‐GACTAGGCAGTGGCGATTCTG‐3′, rev: 5′‐CCACAGACTCCGTTCCACATA‐3′ (Figure S6).

### 2.4 Brevican and Meprin β Cotransfection and Protein Extraction

Wild‐type (wt) and meprin β KO (CRISPR‐Cas9 KO as described above) human embryonic kidney (HEK 293T) cells were chosen for the cotransfection of either brevican and wt meprin β or brevican and the catalytically inactive variant of meprin β (MEP1B E153A). Cells were grown in high‐glucose DMEM (Gibco), supplemented with 10% fetal bovine serum (Gibco), 100 U/mL penicillin (Gibco), 100 μg/mL streptomycin (Gibco), 6 mM glutamine (Gibco), and incubated at 37°C and 5% CO_2_. Transfections were conducted with PEI MAX (Polysciences) following the manufacturer's protocol, using plasmids containing BCAN (ORIGENE), pLHCX, MEP1B (the latter two were generated by our lab; [10]) and MEP1B E153A (a kind gift from the Becker‐Pauly group [21]). The pLHCX plasmid was included as an empty vector control without any transgene. After transfection, proteins were extracted by washing and mechanically detaching the cells with ice‐cold Dulbecco's phosphate buffered saline (Sigma‐Aldrich) and lysing them using cell lysis buffer (150 mM NaCl, 1% Nonidet P‐40, 50 mM TRIS, and 1× protease inhibitor cocktail [complete, Roche applied Science]). Protein concentrations in the lysates were determined using a BCA assay, following the manufacturer's guidelines.

### 2.5 Primary Hippocampal Neuronal Culture

Six‐well plates were prepared by coating with polyornithine (Sigma, P4538) 1:100 in Hank's balanced salt solution (HBSS) (Gibco HBSS without calcium, without magnesium, without phenol red, 14175129) and placed in an incubator for at least 1 h at 37°C. Each well was then washed three times with Millipore water and aspirated before 1 mL of plating medium, which consists of MEM (Gibco, 31095‐031), 10% horse serum (Gibco, 26050070), 0.6% glucose (Fluka, 16646153), and 500 μL glutamine (Gibco, 11539876) was added to each well, and the plate was placed back in the incubator. All preparation instruments were autoclaved before use. Mice were decapitated at P0, and the brain was removed using curved forceps and eye scissors via the foramen magnum and along the sagittal suture. The parietal bones were folded back using forceps, and the brain was transferred into a 3.5 cm petri dish that was filled with HBSS for neurons and placed on ice. Under the dissection microscope, the cerebellum and brain stem were removed, and the hemispheres were separated. The hippocampi were isolated and transferred to tubes with 5 mL HBSS on ice. The tissue was washed three times with ice‐cold HBSS under sterile conditions. Activated trypsin (Gibco, Trypsin–EDTA (0.5%), without phenol red, 15400‐054) was added, and the sample was incubated for 20 min in a water bath at 37°C. DNase (Sigma, DN25100MG) was then added, and incubation continued for an additional 5 min. After that, the tube was removed, and the trypsin was aspirated away. The tissue was then washed twice with HBSS and once more with plating medium. The tissue was then homogenized with 2 mL of plating medium using flamed glass pipettes (Marienfeld) with increasingly narrow tips. The cell suspension was added to a 50 mL falcon tube through a cell sieve (Greiner, EASYstrainer 40 μM, 542040). Live cells were quantified using Trypan blue (Sigma‐Aldrich, T8154) in the Neubauer chamber. One million cells per well of the six‐well plate were seeded in a plating medium. After 30 min, the plating medium was aspirated, and neuron medium, which consisted of Neurobasal Medium A (Gibco, 10888‐022), glutamine (Gibco, 11539876), and B27 supplement (Gibco, 17504044) was pipetted in from the edge of the well. At approximately 2 days in vitro (DIV), 2.5 μL cytosine arabinoside (AraC) per 500 μL of medium was added to each well. At 7 DIV, a medium change with maturation medium consisting of neuronal medium (BrainPhys, 05790) and SM1 Neuronal Supplement (NeuroCult, 05711) took place. This medium change occurred weekly. At ~21 DIV, the culture was ready for the biotinylation assay.

### 2.6 Biotinylation Assay

To assess changes in protein expression on the cell surface of primary neurons, surface biotinylation was performed. To start, cells were placed on ice to inhibit endocytosis, and medium was aspirated. Cells were washed three times with ice‐cold PBS then incubated twice for 20 min at 4°C in a solution containing 0.3 mg/mL sulfo‐NHS‐LC‐LC‐biotin (ThermoFisher, 21335) in PBS to biotinylate surface proteins. Following biotinylation, cells were washed four times with a 50 mM NH_4_Cl solution to neutralize and remove any unbound biotin.

After a final wash with PBS, cells were lysed with RIPA buffer (150 mM NaCl, 10 mM TRIS–HCl, 1 mM EDTA, 1% Triton X‐100, 0.1% SDS, 0.1% sodium deoxycholate), and total protein concentration was measured to ensure equal protein loading. Equal protein amounts were incubated overnight at 4°C with 30 μL NeutrAvidin agarose beads (ThermoFisher, 29201) for specific binding and precipitation of biotinylated proteins. The beads were subsequently washed twice with RIPA buffer, and bound proteins were eluted by boiling at 95°C in SDS loading buffer. Finally, proteins were analyzed by SDS‐PAGE followed by western blotting to quantify changes in surface protein expression.

### 2.7 SDS‐PAGE and Western Blotting

For western blotting, 20 μg of lysate was loaded. S2 fraction samples were incubated with Chondroitinase ABC from *Proteus vulgaris* (Sigma‐Aldrich C3667) for 30 min at 37°C. Then, for BCAN and meprin β cotransfection, synaptosome or S2 fraction samples (pooled Hippocampi and single Cortices) were prepared in 1× SDS loading buffer (4× Roti‐Load, Carl Roth, Germany) and boiled at 95°C. Protein extracts were separated by SDS‐PAGE, transferred onto nitrocellulose membranes (Amersham Hybond ECL), and then blocked in 5% (w/v) milk powder in TBST. The samples were analyzed for the expression of several different synaptic proteins using the following antibodies: NMDAR1a (Invitrogen 32‐0500), NMDAR2a (Santa Cruz sc‐515148), NMDAR2b (Invitrogen MA1‐2014), AMPAR 2/3/4 (Cell Signaling 2460), Brevican (Invitrogen PA5‐31444), α‐tubulin (Invitrogen 62204), GAPDH (Invitrogen PA1‐987), anti‐HA‐tag (3F10 Roche 11867427001), anti‐MYC‐tag (Cell Signaling 2278), and anti‐strep‐tag (Qiagen 34850). To visualize multiple proteins at once, we utilized fluorescent secondary antibodies. Depending on the primary antibody's species specificity, we paired the secondary antibodies accordingly with a combination of the following dyes: IRDye800CW and IRDye680CW (both for rabbit and mouse), along with StarBright Blue 520 and StarBright Blue 700. This approach allows us to see multiple proteins of interest efficiently on a single membrane.

### 2.8 Immunofluorescence (IF) Staining of Brain Sections

Mouse brains were perfused with 4% paraformaldehyde (PFA) and subsequently embedded in a tissue freezing medium (Leica). Sagittal sections of 30 μm thickness were prepared using a CM3050S cryostat (Leica) and transferred into 48‐well plates containing cryoprotective solution (25% glycerol, 25% polyethylene glycol, and 50% PBS). Sections were stored at 4°C until further processing. For IF, cryosections of mouse brains were postfixed in 4% paraformaldehyde overnight and processed as free‐floating slices. All washing steps were carried out using 0.01 M phosphate buffered saline (PBS). To block nonspecific binding and permeabilize the tissue, sections were incubated for 2 h at room temperature in PBS containing 0.8% Triton X‐100 and 7% normal donkey serum (Dianova, 017‐000‐121).

Primary antibody incubation was performed for 3 days at 4°C in PBS supplemented with 2% bovine serum albumin (IgG‐free, protease‐free; Jackson ImmunoResearch, 001‐000‐161, via Dianova), 0.05% sodium azide, and 0.3% Triton X‐100. The following primary antibodies were applied: goat anti‐meprin β (1:40, Thermo Fisher Scientific, PA5‐47474), rabbit anti‐VGLUT1/2 (1:500, Synaptic Systems, 135503), and an alpaca‐derived FluoTag‐X2 anti‐PSD95‐AZDye 568 nanobody (1:200, NanoTag/Synaptic Systems, N3702‐AF568‐L).

Following primary incubation, sections were washed with PBS and incubated for 2 h at room temperature with the respective secondary antibodies and DAPI (0.5 μg/mL, AppliChem, A4099,0005) in PBS containing 2% bovine serum albumin and 0.05% sodium azide. Secondary antibodies used were IRDye 680RD donkey anti‐goat IgG (1:200, Li‐COR Biosciences, 925‐68074) and Alexa Fluor 488‐conjugated donkey anti‐rabbit IgG (H + L) (1:200, Jackson ImmunoResearch, 711‐545‐152, via Biozol).

After final washes in PBS, sections were mounted using Fluoromount‐G (Thermo Fisher Scientific) and stored protected from light until further processing for microscopy.

### 2.9 Confocal Imaging of CA3 Hippocampal Sections

Stained sagittal brain slices (thickness: 30 μm) from the CA3 region of the murine hippocampus were imaged using a Zeiss LSM 710 confocal laser scanning microscope. The following acquisition settings were used: DAPI (pseudo‐gray) was excited at 405 nm (pinhole 41.9 μm, gain 850), VGLUT (pseudo‐green, Alexa Fluor 488) at 488 nm (pinhole 51.4 μm, gain 750), PSD‐95 (pseudo‐blue, Alexa Fluor 568) at 543 nm (pinhole 59.4 μm, gain 900), and meprin β (pseudo‐red, IRDye 680 detected via the Atto680 channel) at 633 nm (pinhole 67.8 μm, gain 1200). Pixel dwell time was 54.93 μs, frame size 1912 × 1912 or 4096 × 4096 pixels (necessary for synapse‐imaging), and line averaging was set to 16. Images were processed in ImageJ by subtracting background (rolling ball radius = 50 pixels), applying Gaussian blur (1 px), and slightly enhancing contrast. Scale bar: 20 μm.

### 2.10 Electrophysiology

Mice were deeply anesthetized with 4 vol% isoflurane until loss of involuntary reflexes. Then they were quickly decapitated, the brain immediately removed and put into the ice‐cold (4°C) choline‐based artificial cerebrospinal fluid (cACSF) perfused with carbogen (95% oxygen [O_2_] and 5% carbon dioxide [CO_2_]). This cutting solution contained (in mM): NaCl (87), KCl (2.5), NaH_2_PO_4_ + H_2_O (1.25), choline chloride (37.5), NaHCO_3_ (25), and D‐glucose (25), CaCl*2H_2_O (0.5), MgCl_2_ (7) and at a pH of 7.4. Next, the brain was placed on the stage of a vibratome (VT1200 S, Leica) in an orientation to cut horizontal brain slices with a thickness of 400 μm. Slices containing the distinctive hippocampal structures were selected and put into a storage chamber filled with normal, carbonated ACSF containing (in mM): NaCl (125), NaH_2_PO_4_ (1.25), KCl (2.5), NaHCO_3_ (25), D‐glucose (25), CaCl_2_*2H_2_O (2), MgCl_2_ (1) and kept at room temperature. Slices rested in ACSF for at least 40 min before individual slices were transferred onto a multi‐electrode array (MEA) chip of a two‐chamber MEA system (MEA2100 System, Multi‐Channel Systems MCS GmbH), which was constantly perfused with normal ACSF at 32°C. Each glass MEA chip consisted of 60 electrodes, each with a diameter of 30 μm and spaced with a 200 μm interelectrode distance (60MEA200/30iR; Multi‐Channel Systems MCS GmbH). Each brain slice was placed in the most optimal manner to have well‐positioned stimulating electrodes in the CA3 region and recording electrodes in the CA1 region of the hippocampus. A platinum grid was placed on top of the slices to stabilize their position. Slices were allowed to settle on the MEA chip for a minimum of 30 min before any recordings began. The electrophysiological recording protocols were generated and applied by Multi Channel Experimenter 2.2 software (Multi Channel Systems MCS GmbH) using a 50 kHz sampling rate and a Butterworth highpass 2.0 Order Filter with a 200 Hz cutoff.

Input/output (I/O) curves of extracellular recorded field postsynaptic potentials (fEPSPs) were generated by electrical stimulation of one MEA electrode starting with a voltage pulse of 500 mV at a stimulus duration of 100 μs, then gradually increasing the pulse intensity by +500 mV until a maximum stimulation intensity of 5000 mV was reached. The duration between each voltage pulse stimulation was 40 s.

The paired‐pulse protocol used a stimulation intensity that resulted in an fEPSP equal to approximately 30% of the maximum evoked fEPSP amplitude from the input/output curve protocol. Paired pulses had a 50 ms interstimulus interval.

To test the strength of synaptic long‐term potentiation (LTP), fEPSPs were recorded with the same stimulus intensity selected for the paired‐pulse protocol, on a slicewise basis. Baseline stimulations for LTP were applied for 10 min, consisting of one stimulation per minute. Then, LTP was induced by applying a high‐frequency stimulation (HFS) at 100 Hz for 1 s, followed by 60 min of baseline stimulations every 60 s. The level of HFS‐induced LTP was analyzed by normalizing the mean amplitudes from the last 10 min of baseline recordings after HFS (50–60 min) to the mean of the amplitude of the initial 10 min baseline recordings before HFS. These data were compared to recordings against the independent control pathway that was generated by a second stimulation electrode located in the direction of the subiculum, as it cannot generate long‐term plasticity changes in CA1. The Multi‐Channel Analyzer 2.2 (Multi‐Channel Systems MCS GmbH) software was used to record the peak value of each fEPSP amplitude.

All electrophysiological data were analyzed using the Multi‐Channel Analyzer 2.2 software, Microsoft Office Excel (Microsoft), and GraphPad Prism (GraphPad Software).

### 2.11 TMT N‐HUNTER Proteomics

Samples (*n* = 3 per genotype) were processed according to TMT HUNTER workflow [22]. Precipitated proteins were reconstituted in 1 mL (1% SDS, 1× complete EDTA‐free Protease Inhibitor Cocktail (Roche), 50 mM HEPES pH 8). Protein concentration was determined using Pierce BCA Protein Assay Kit (Thermo). One hundred micrograms of proteins of each sample were reduced and alkylated by mixing with 24 mM TCEP, 80 mM Chloroacetamide for 1 h at 25°C in the dark. First clean‐up was performed with SP3 beads (1:20 w/w protein to beads ratio) and ethanol (EtOH) added to 50% (v/v) with mixing for 15 min at 25°C to induce protein binding. Then, supernatant was removed and beads were washed three times with 80% EtOH. To commence with TMT labeling, beads were resuspended in 22.5 μL 6 M Guanidine hydrochloride, 30 μL 0.5 M HEPES (pH 8), and 4.5 μL 125 mM TCEP at 25°C for 30 min. Isobaric tandem mass tags (0.8 mg TMT6plex reagents (Thermo)) were dissolved in 57 μL EtOH by occasional vortexing for 5 min. Samples were separated with TMT6plex reagents and labeling was performed by adding a label to each beads‐bound protein sample (one tag per sample) and mixing for 1.5 h at 25°C in the dark. Labeling was stopped by adding 8 μL 8% hydroxylamine for 30 min at 25°C. A second clean‐up was done by combining each group of samples and adding EtOH to 80% for 15 min at 25°C. Then, supernatant was removed, and beads were washed three times with 80% EtOH. Beads were spun down to remove any remaining liquid then reconstituted in 200 mM HEPES (pH 8). Digestion was performed with trypsin (1:50 enzyme to protein ratio) overnight at 37°. Ten microliters of the supernatant (corresponding to 15 μg of protein) was taken as a pre‐HUNTER and the rest (HUNTER) was labeled with undecanal by adding EtOH to 40%, 12 μL undecanal 97% and sodium cyanoborohydride to 30 mM final concentration for 1 h at 37°C. Labeled peptide solution was then acidified with Trifluoroacetic acid (TFA) to pH 3 and undecanal clean‐up was performed using Sep‐Pak tC18 Cartridges 1 cc (Waters). Conditioning and equilibration were performed with methanol and 40% EtOH, 0.1% TFA, respectively. Samples were added and 0.1% TFA and flow through was collected. Collected fractions were dried in a Speedvac for 3 h then freeze‐dried overnight. Desalting of the Pre‐HUNTER and HUNTER samples was done with 10 μL Pierce C18 Spin Tips (Thermo) and Sep‐Pak C18 Cartridges 1 cc (Waters), respectively. Eluted peptides were vacuum dried and stored at −20°C until analysis.

Samples were resuspended in 3% ACN with 0.1% TFA and injected for LC–MS analysis. Chromatographic separation was performed on a Dionex U3000 nanoHPLC system equipped with an Acclaim pepmap100 C18 column (2 μm particle size, 75 μm × 500 mm) coupled online to a mass spectrometer. The eluents used were eluent A: 0.05% FA, eluent B: 80% ACN + 0.04% FA. For pre‐HUNTER samples, the separation was performed over a programmed 90 min run. Initial chromatographic conditions were 4% B for 2 min followed by linear gradients from 4% to 50% B over 60 min, then 50%–90% B over 5 min, and 10 min at 90% B. Following this, an interrun equilibration of the column was achieved by 13 min at 4% B. For HUNTER samples, the separation was performed over a programmed 220 min run. Initial chromatographic conditions were 4% B for 2 min followed by linear gradients from 4% to 50% B over 180 min, then 50%–90% B over 10 min, and 13 min at 90% B. Following this, an interrun equilibration of the column was achieved by 15 min at 4% B. A constant flow rate of 300 nL/min was employed. Wash runs were performed between each sample injection. Data acquisition following separation was performed on a QExactive Plus (Thermo). Full scan MS spectra were acquired (350–1400 *m*/*z*, resolution 70 000) and subsequent data‐dependent MS/MS scans were collected for the 10 or 15 most intense ions (Top10 or Top15) for pre‐HUNTER and HUNTER, respectively, via HCD activation at NCE 33 (resolution 17 500). Dynamic exclusion (40 s duration) and a lock mass (445.120025) were enabled.

Raw data were analyzed against a database containing a reviewed Uniprot mouse proteome ( *Mus musculus* ) (17 530 sequences) and common contaminants (cRAP). The search was performed on Proteome Discoverer 2.2 using a SequestHT search engine with 10 ppm and 0.02 Da precursor and fragment ion tolerances, respectively. Digestion with Trypsin_R (semi) with a max of two missed cleavages was applied. Oxidation of methionine (15.995 Da), acetyl (42.011 Da), and TMT6plex (229.163 Da) at the peptide N‐terminus was set as a dynamic modification. Carbamidomethylation (57.02146 Da) on cysteine and TMT6plex on lysine was set as a static modification. For labeling efficiency calculations, TMT6plex on lysine was set as a dynamic modification. Strict parsimony criteria have been applied, filtering peptides and proteins at 1% FDR.

### 2.12 Morris Water Maze Paradigm Test

For behavior analysis, 8‐month‐old mice were tested (*n* = 7 animals/group). Spatial learning and memory were assessed by the Morris' Water Maze hidden platform task performed as previously described with minor modifications [14]. Briefly, the water maze (diameter 120 cm) was filled with clear water (21°C–22°C) 1 cm above the platform. Prominent symbols around the maze provided abundant extra‐maze cues. The platform stayed in the same quadrant from days 1 to 4 and the animals were released from four different positions at the pool perimeter. Mice performed four trials per day on 4 consecutive days with a maximum length of 90s. Each day the animals were released into the water from different locations outside the platform (d1—north, south, east, west; d2—W‐E‐S‐N; d3—S‐W‐N‐E; d4—E‐N‐W‐S). If mice did not find the platform within the given time, they were guided to the platform. Mice were allowed to stay on the platform for 10 s to memorize the surrounding cues. On the fifth experimental day, a probe trial (60 s) without the platform was performed. Basal motor activity was evaluated by swim speed (Figure S5). Learning was assessed by measuring the escape latency to find the platform. Memory capabilities were characterized by the number each mouse crossed the former platform location at probe trial and the latency to reach the location. To further assess the ability to memorize the location of the platform, the time spent in the right quarter of the platform was measured. For vision abilities, a visible platform task was done after the learning assessment at day 6. It consisted of three trials in a row starting opposite to the platform, which was indicated by a table tennis ball 15 cm above the platform. No mouse failed this task, showing that all tested mice were capable of seeing the cues (not shown).

### 2.13 Monitoring of Behavior

A computerized video recording system registered the moving path and duration in water maze tests automatically. The hardware consisted of an IBM‐type AT computer combined with a video digitizer and a CCD video camera. The software used for data acquisition and analysis was EthoVision XT release 8.0 (Noldus Information Technology, Utrecht, the Netherlands).

### 2.14 Statistical Analysis and Illustrations

All graphs and statistical analyses were prepared using GraphPad Prism 8 software (La Jolla, CA). Western blots were quantified by densitometry analysis using ImageJ v.1.52 (NIH, USA). The individual statistical tests were described in the respective figure descriptions (ns: *p* > 0.05; *p ≤ 0.05; **p ≤ 0.01; ***p ≤ 0.001). The figures were created with Microsoft PowerPoint, BioRender.com, and/or GraphPad.

## 3 Results

### 3.1 Generation of a Targeted Meprin β‐Overexpressing Mouse Model

We previously described a learning and memory‐related effect of meprin β in the brain beyond its role in APP processing [14]. Behavioral analysis revealed that meprin β KO animals outperformed their wild‐type littermates, suggesting an enhanced ability for memory consolidation. To further investigate this, we established a mouse model that overexpresses meprin β in neurons of the hippocampus and cortex, the brain regions primarily responsible for learning and memory formation.

**FIGURE 1:**
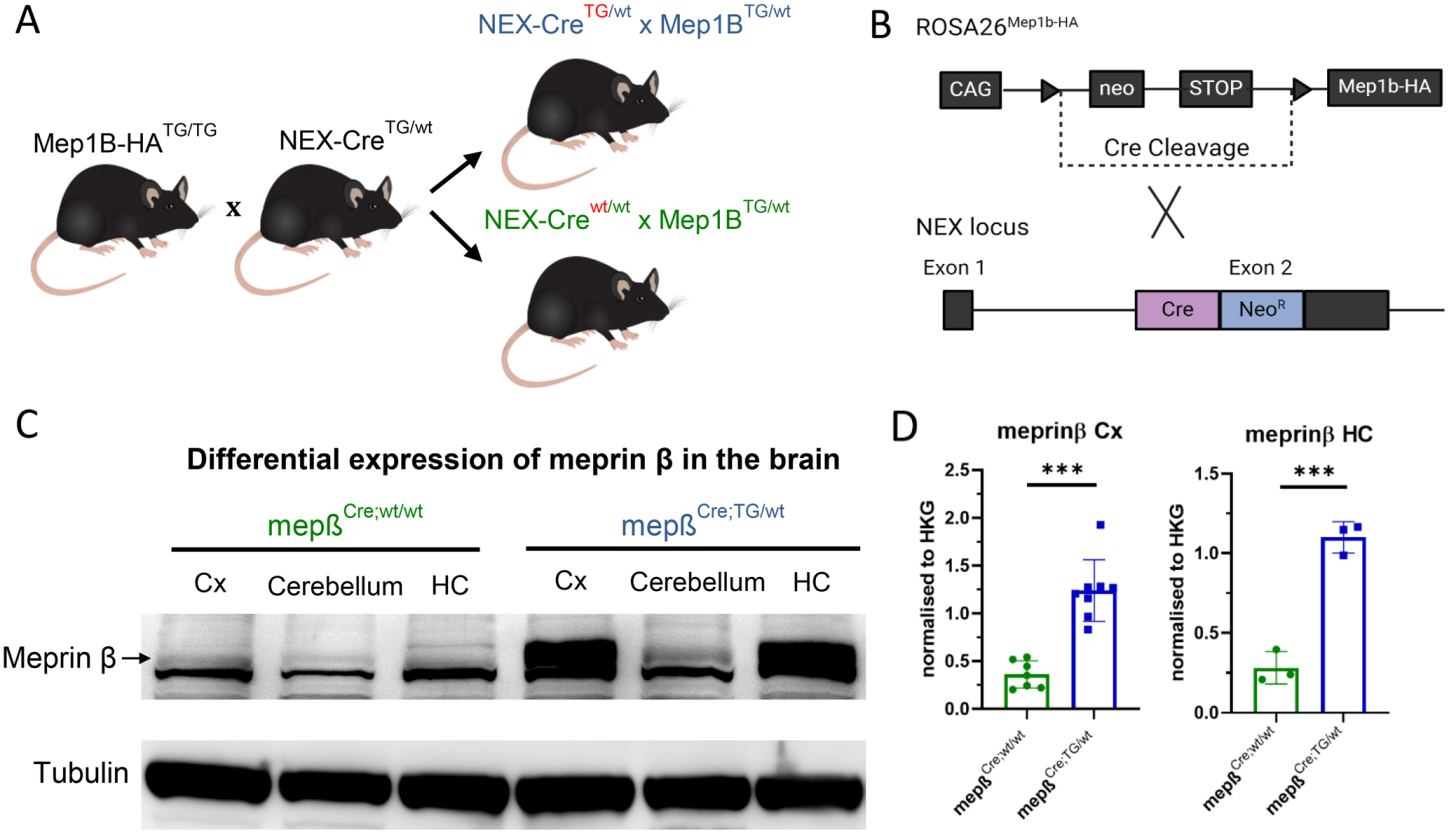
Generation of a targeted meprin β overexpression in cortical and hippocampal neurons. (A) Homozygous transgenic *Rosa26* ^*Mep1b‐HA*^ mice were crossed with heterozygous *NEX* ^*Cre*^ mice, yielding *NEX* ^*Cre;wt/wt*^ × *Rosa26* ^*Mep1b‐HA*^ (mepβ^Cre;wt/wt^) and *NEX* ^*Cre;TG/wt*^ × *Rosa26* ^*Mep1b‐HA*^ (mepβ^Cre;TG/wt^) offspring. (B) For cortex‐ and hippocampus‐specific overexpression of meprin β, we employed the NEX‐Cre mouse model, where Cre recombinase expression is directed by NEX regulatory sequences. This was achieved by substituting the NEX coding region with a Cre cassette via homologous recombination. (C) Western blot analysis across different brain regions of *NEX* ^*Cre;wt/wt*^ × *Rosa26* ^*Mep1b‐HA*^ (wild‐type) and *NEX* ^*Cre;TG/wAAAAAAAAAAAAAAAAt*^ × *Rosa26* ^*Mep1b‐HAX*^ (meprin β‐overexpressing) of 8‐month‐old mice showed elevated meprin β expression specifically inside the cortex and hippocampus of *NEX* ^*Cre;TG/wt*^ × *Rosa26* ^*Mep1b‐HA*^ transgenic animals. (D) Densitometric quantification of meprin β in cortex and hippocampus of *NEX* ^*Cre;wt/wt*^ × *Rosa26* ^*Mep1b‐HA*^ *and NEX* ^*Cre;TG/wt*^ × *Rosa26* ^*Mep1b‐HA*^ (overexpressing) mice from several experiments normalized to the housekeeping gene (HKG) tubulin.

To generate a targeted meprin β‐overexpression in the cortex and hippocampus, we utilized the previously described NEX‐Cre mouse model where the Cre recombinase is expressed under control of the regulatory sequences of NEX, a gene that encodes a neuronal basic helix–loop–helix protein. The coding region of NEX (exon 2) was replaced by a Cre cassette using homologous recombination (Figure 1B) [19]. Animals harboring this mutation were crossed with meprin β knock‐in animals as described in [20]. The knock‐in was generated by cloning murine meprin β cDNA into STOP‐EGFP‐ROSA‐CAG targeting vector and inserting it into the Rosa26 locus of embryonic stem cells by homologous recombination.

This breeding resulted in heterozygous meprin β‐overexpressing offspring (mepβ^Cre;TG/wt^) with consistent expression of meprin β specifically in the hippocampus and cortex (Figure 1A). Figure 1C shows the expression of meprin β in wild‐type mice and the novel mepβ^Cre;TG/wt^ mouse model: apart from low endogenous meprin β expression in wild‐type animals and the cerebellum of mepβ^Cre;TG/wt^ mice, we can see a significant increase in meprin β expression in the cortex and hippocampus of mepβ^Cre;TG/wt^ animals.

Densitometric analysis of western blots revealed that meprin β was overexpressed approximately threefold in the cortex and fivefold in the hippocampus of transgenic mice compared to endogenous meprin β levels in wild‐type controls (Figure 1D).

### 3.2 Meprin β Overexpression in Cortex and Hippocampus Leads to Impairment in Learning and Memory

**FIGURE 2:**
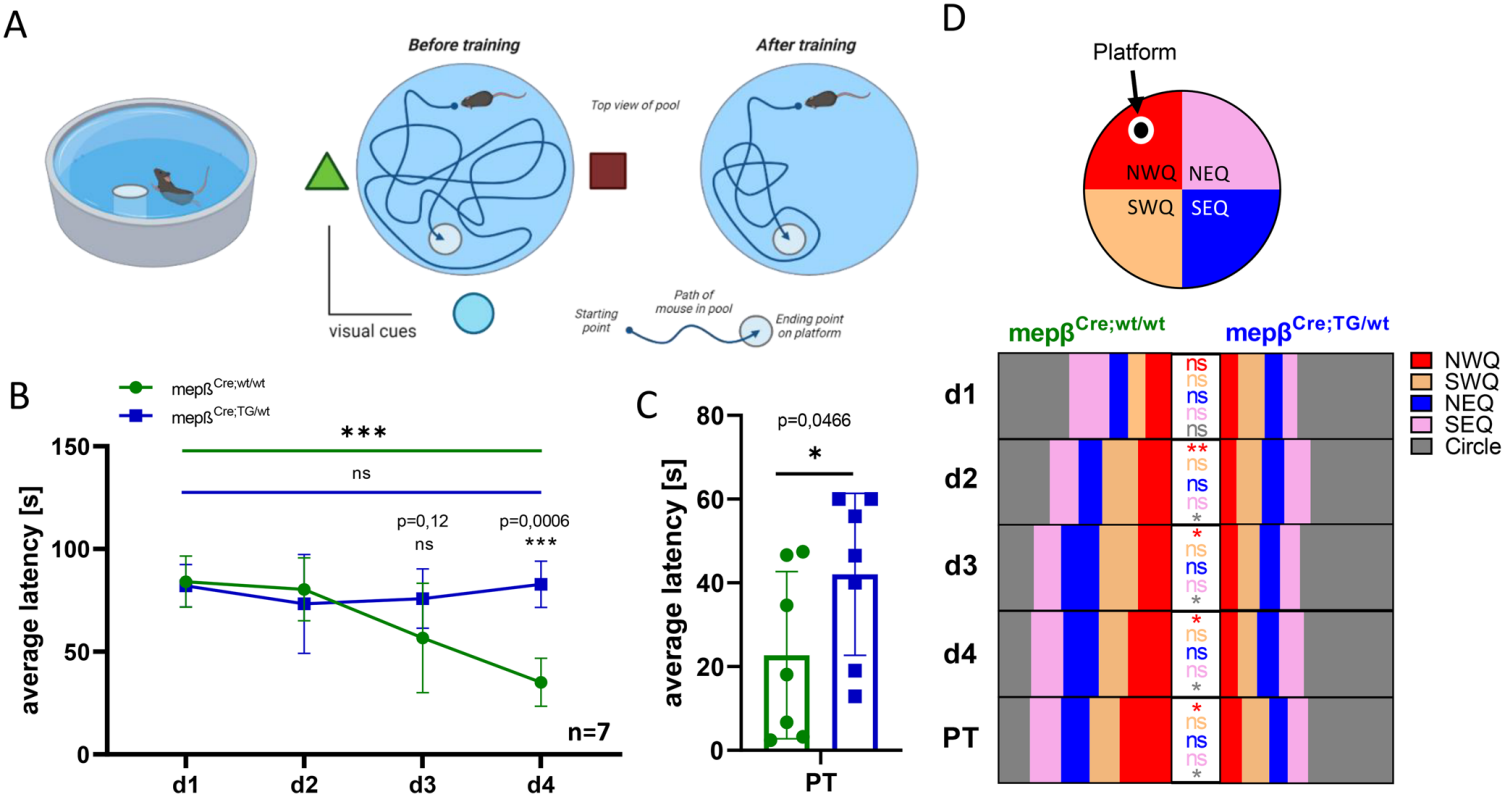
Meprin β overexpression leads to cognitive impairment. Spatial learning and memory tests utilizing the Morris water maze (MWM) paradigm tests were carried out on 8‐month‐old meprin β‐overexpressing mice and their wild‐type counterparts (*n* = 7). (A) Schematic overview on the Morris' Water Maze paradigm test: The platform is submerged under water, but the animals have visual cues on the wall of the water tank for navigation. Training lasts for 4 days, with four trials per day. (B) The average escape latency in each trial was measured for the four training days. On day 4, the meprin β‐overexpressing animals showed significant learning deficit compared to mepβ^Cre;wt/wt^ mice (*p* = 0.006). Data shown are the mean ± SEM of four different trials performed on each day. Statistical analysis was performed using an unpaired parametric *t*‐test. (C) On probe trial day, the platform was removed, and the latency to reach the former platform location was measured. Similar to the final training day, significant cognitive deficits can be observed for meprin β‐overexpressing mice compared to mepβ^Cre;wt/wt^ animals (*p* = 0.0466). Statistical analysis was conducted using an unpaired Mann–Whitney *U* test due to the data's nonnormal distribution, as determined by the Shapiro–Wilk test. (D) The water tank was divided into four equal quadrants, with the platform located in the NWQ quadrant. The time spent by the animals in each of the four quadrant or circling the water tank was measured. It is observable that after the second day, the mepβ^Cre;wt/wt^ animals spent significantly more time in the correct quadrant, although this difference is not directly reflected in latency finding the platform. Additionally, after the second day, mepβ^Cre;wt/wt^ animals spent significantly less time circling the platform. Statistical analysis was performed using an unpaired parametric *t*‐test.

After generating the mouse model, we aimed to compare learning and memory performance in a behavioral experiment between mepβ^Cre;wt/wt^ and mepβ^Cre;TG/wt^ animals. To investigate this, we utilized the Morris' Water Maze paradigm test (Figure 2A).

Clear differences between mepβ^Cre;TG/wt^ and mepβ^Cre;wt/wt^ animals emerged as early as the third day of training. Mepβ^Cre;wt/wt^ animals could locate the platform significantly faster by the fourth training day, whereas meprin β‐overexpressing mice showed no evidence of learning (Figure 2B). The time required to locate the platform remained constant from the first to the last training day for mepβ^Cre;TG/wt^ mice, indicating a pronounced learning impairment. Furthermore, during the probe trial, these animals took significantly longer to find the former platform location compared to mepβ^Cre;wt/wt^ counterparts (Figure 2C). Although the time to locate the platform decreased for mepβ^Cre;TG/wt^ animals during the probe trial compared to the fourth training day, suggesting minimal learning, this effect was likely coincidental: analysis of the time spent in the correct quadrant of the water tank revealed that meprin β‐overexpressing mice failed to exhibit directed swimming toward the platform's original location. In contrast, mepβ^Cre;wt/wt^ animals displayed a clear preference for the correct quadrant, indicating effective learning and spatial memory already on day 2. Additionally, mepβ^Cre;wt/wt^ animals spent significantly less time circling the water tank compared to meprin β‐overexpressing animals (Figure 2D).

In summary, transgenic mice exhibit substantial learning impairments in the Morris' Water Maze paradigm test. Together with prior findings showing beneficial effects of meprin β knockout mice on learning [14], these results suggest that meprin β may play a significant role in learning and behavioral processes.

### 3.3 Meprin β Localizes to Axons and Synapses and Colocalizes With PSD‐95 in Overexpressing Neurons

**FIGURE 3:**
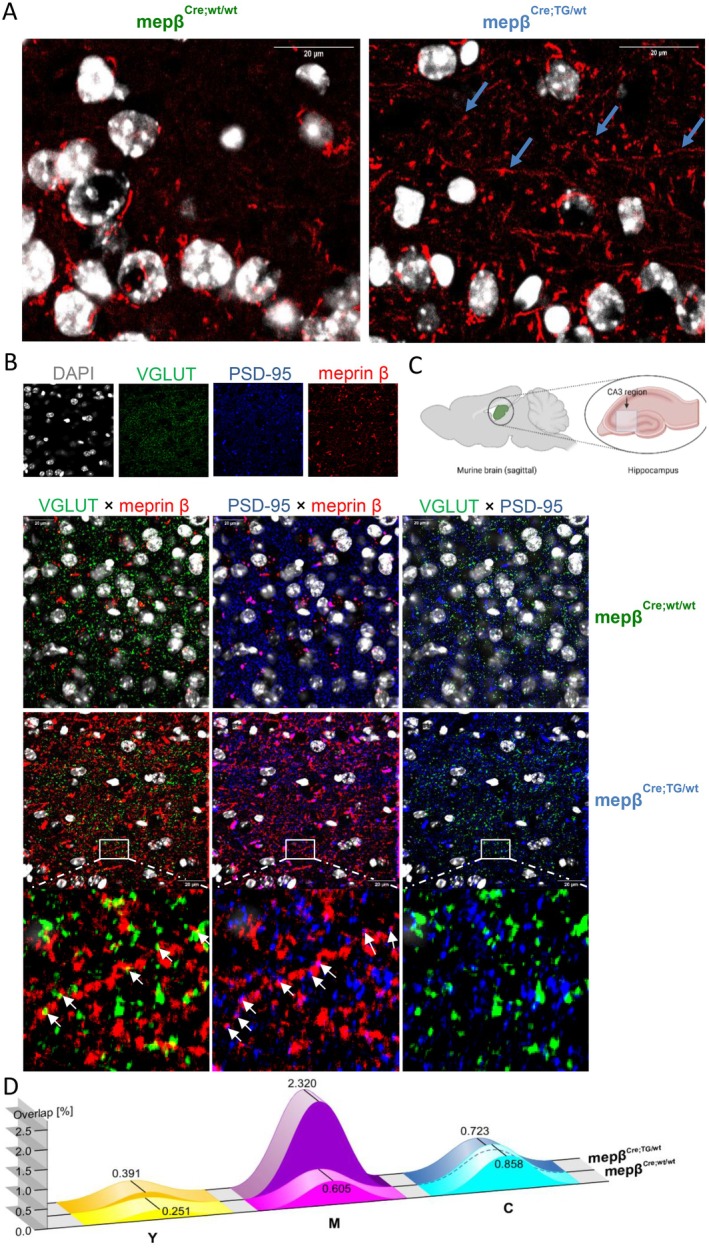
Subcellular localization of meprin β in hippocampal neurons of wild‐type and meprin β‐overexpressing mice. Sagittal brain slices (thickness: 30 μm) from the CA3 region of the murine hippocampus were imaged using a Zeiss LSM 710 confocal laser scanning microscope. (A) Representative confocal images show meprin β (red) and DAPI (gray) staining in wild‐type (mepβ^Cre;wt/wt^) and meprin β‐overexpressing (mepβ^Cre;TG/wt^) animals, revealing increased signal and extended axonal and synaptic distribution in the latter indicated by arrows. (B) Single‐channel and merged images of DAPI (gray), VGLUT (green), PSD‐95 (blue), and meprin β (red) in wild‐type and overexpressing animals. Merged signals appear as yellow (meprin β + VGLUT), magenta (meprin β + PSD‐95), and cyan (VGLUT + PSD‐95). Zoomed‐in views highlight overlapping patterns, indicated by arrows. (C) Schematic diagram of the CA3 region indicating the anatomical site of image acquisition. (D) Quantification of colocalization was performed in ImageJ by splitting RGB channels, thresholding (triangle method), and using the “Image Calculator” to determine overlap area fractions (in %). Increased meprin β + PSD‐95 colocalization was observed in overexpressing animals, with no crucial changes in the other combinations.

Using the meprin β‐overexpressing mouse model (mepβ^Cre;TG/wt^) that overexpresses meprin β in cortical and hippocampal neurons, we employed confocal microscopy to examine its distribution in sagittal brain slices, focusing on the CA3 region of the hippocampus (Figure 3C). This area was selected based on findings showing that meprin β‐overexpressing mice exhibit impairments in spatial learning in the MWM. In wild‐type animals, the meprin β signal was primarily perinuclear, likely reflecting retention in the endoplasmic reticulum. In contrast, overexpressing mice showed stronger staining intensity throughout the soma, with additional localisation in axons and synaptic regions (Figure 3A). To assess synaptic distribution more specifically, we performed costaining with the presynaptic marker VGLUT and the postsynaptic marker PSD‐95 (Figure 3B). The overlap between meprin β and VGLUT appeared as yellow, while the overlap between meprin β and PSD‐95 was visualized as magenta, and the overlap between VGLUT and PSD‐95 as cyan. While the yellow and cyan signals did not differ substantially between wild‐type and overexpressing animals, the magenta signal was notably more prominent in the overexpression condition, indicating an increased postsynaptic presence of meprin β. These findings align with our behavioral data suggesting a postsynaptic mechanism underlying the cognitive impairments observed in these animals.

Staining with an anti‐HA antibody, which selectively detects the transgenic overexpressed meprin β, further supported its presence at axonal and synaptic sites in overexpressing neurons and produced a sharper signal than the conventional antibody. Both HA‐tag and secondary antibody‐only control stainings are presented in Supplementary Figure 1.

### 3.4 Meprin β‐Overexpressing Animals Show Significantly Impaired Hippocampal LTP

**FIGURE 4:**
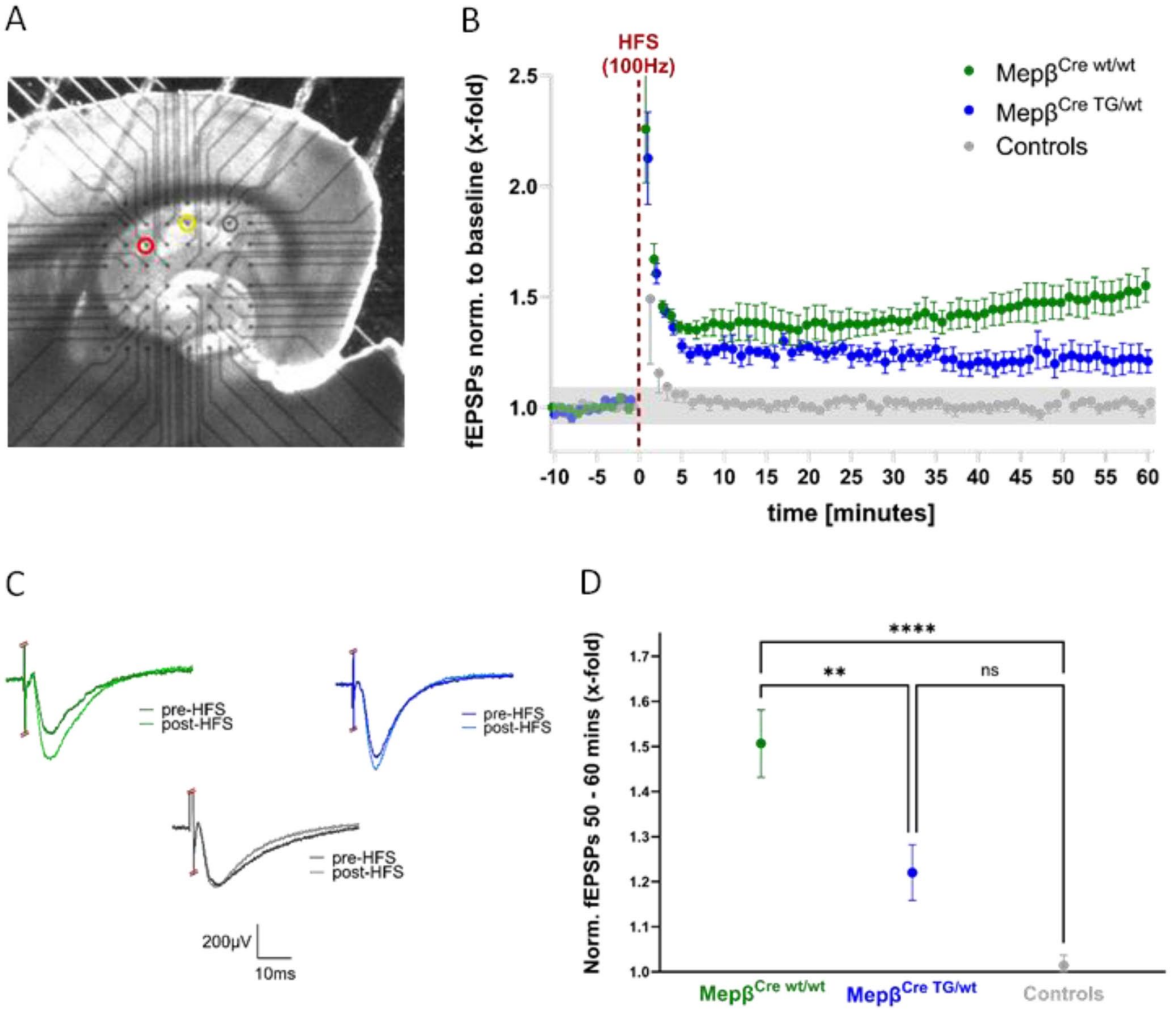
Meprin β‐overexpressing mice show an impaired hippocampal LTP. Mepβ^Cre;wt/wt^ mice achieve a level of LTP that is significantly larger than both the control recordings and the mepβ^Cre;TG/wt^ recordings. (A) Example of the location of stimulating electrode (red), recording electrode (yellow), and stimulating electrode of the independent control pathway (gray) along the Schaffer Collaterals. (B) LTP was induced at the Schaffer collaterals using a 100 Hz high‐frequency stimulation (HFS) protocol and recorded in area CA1 using a microelectrode array (MEA) in mepβ^Cre;wt/wt^ and mepβ^Cre;TG/wt^ mice. Green dots represent the mepβ^Cre;wt/wt^ recordings, blue dots represent mepβ^Cre;TG/wt^ recordings, and the gray dots represent the recordings taken from the independent control pathway. Graph shows mean ± SEM of the relative strength of potentiation of fEPSPs. Mepβ^Cre;wt/wt^ *n* = 7 slices from 6 mice. Mepβ^Cre;TG/wt^ *n* = 7 slices from four mice. Control pathway *n* = 6 slices from 5 mice. (C) Representative fEPSP traces from both mepβ^Cre;wt/wt^ (green) and mepβ^Cre;TG/wt^ (blue) genotypes along with the traces from the independent control pathway (gray). The darker of each color shows fEPSPs before the 100 Hz stimulation, the lighter of each color shows fEPSPs 60 min after the tetanic stimulation. (D) The relative strength of potentiation of the fEPSPs recorded at 50–60 min after the 100 Hz stimulation shows that the mepβ^Cre;TG/wt^ genotype has a significantly lower LTP than mepβ^Cre;wt/wt^ as well as revealing no significant difference compared to the independent control pathway (*p* = 0.05). Statistical analysis using one‐way ANOVA with post hoc Tukey's multiple comparisons test. Graph shows mean ± SEM.

As we have detected severe learning and memory differences in meprin β‐overexpressing mice and additionally a higher presence at the postsynapse, we tested the impact of meprin β overexpression on long‐term synaptic plasticity, a cellular model of learning and memory. Therefore, we carried out electrophysiological long‐term potentiation (LTP) recordings of acute mepβ^Cre;wt/wt^ and mepβ^Cre;TG/wt^ mouse hippocampal tissue slices by use of the MEA. A representative slice on the MEA chip can be seen in Figure 4A. First, we recorded baseline extracellular field excitatory postsynaptic potentials (fEPSPs) in the CA1 region at a stimulation intensity that evoked approximately 30% of the maximum evoked response from the I/O curve on an individual slice basis.

Following 10 min of stable baseline recordings, we applied electrical high‐frequency stimulation (HFS) of 100 Hz for 1 s at the Schaffer collaterals in area CA3. As an additional, independent control input, HFS of equal intensity was also exerted from another, second pathway onto CA1. As expected, this independent, second input failed to reveal any potentiation of synaptic fEPSPs in area CA1 (Figure 4B, gray dots). Representative evoked fEPSP traces from before and after the HFS for mepβ^Cre;wt/wt^ (green) and mepβ^Cre;TG/wt^ (blue) slices, and the control stimulation pathway (gray), can be seen in Figure 4C. Statistical analysis of the fEPSPs was carried out based on the mean potentiation values of the last 10 min of the 60 min recording period following HFS for each slice, relative to each of their baseline fEPSP amplitudes (Figure 4D). As expected, and shown in Figure 4D, LTP was successfully induced at CA1 synapses from mepβ^Cre;wt/wt^ mice. The LTP induction after HFS in mepβ^Cre;TG/wt^ mice, however, was significantly impaired. Meprin β overexpression resulted in a significantly lower hippocampal potentiation compared to the mepβ^Cre;wt/wt^ mice, as well as having no statistical difference from the independent control pathway (Figure 4D).

In summary, mepβ^Cre;TG/wt^ mice have a significantly impaired LTP in the CA3‐CA1 pathway, which indicates functional impairment of the synaptic properties normally inducing NMDAR‐dependent type of LTP. This further suggests potential behavioral deficits in hippocampal‐dependent learning and memory tasks in these animals, and it might link those changes to functional alterations of postsynaptic AMPA and/or NMDARs.

### 3.5 Glutamatergic Receptor Expression at the Synapse Is Not Altered in Meprin β Overexpressing Animals

Meprin β commonly exhibits peak activity on the outer cellular membrane of the cell and in its soluble form, within the ECM [23]. Since meprin β was associated with the postsynapse, we focused on membrane‐bound key mediators in postsynaptic transmission. The two main regulators of synaptic excitatory transmission are the glutamatergic receptors, NMDA and AMPA. While NMDA receptors are required for LTP establishment [24], AMPA receptors are responsible for baseline synaptic transmission [24]. We examined both receptors by SDS‐PAGE and western blotting to explore the potential variability in receptor expression between mepβ^Cre;wt/wt^ and meprin β‐overexpressing mice, which might contribute to the establishment of cognitive impairment.

**FIGURE 5:**
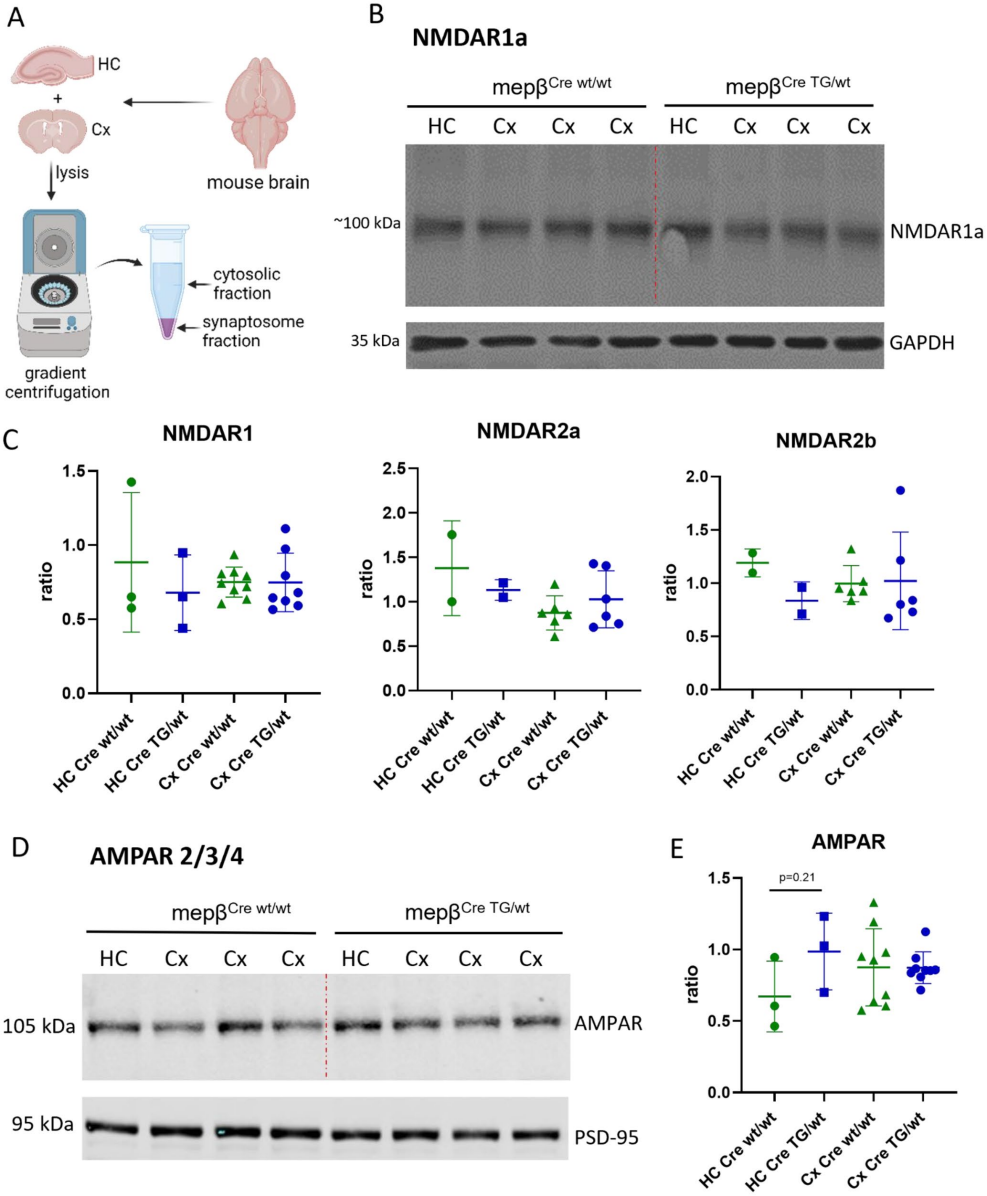
Meprin β overexpression has no effect on NMDA‐ or AMPA‐receptor expression at cortical or hippocampal synapses. Due to low yield in isolating the hippocampal synaptosome fraction, samples from two to three animals of the same genotype were pooled. Thus, each data point for the hippocampus represents multiple animals. For statistical analysis, unpaired Student's *t*‐test was used. (A) Schematic overview of the synaptosome isolation of murine cortex and hippocampus. We used nine mice in three independent experiments. (B) Western blot targeting NMDAR subunit 1 and GAPDH for normalization. The dotted red line separates the mepβ^Cre;wt/wt^ from the transgenic mepβ^Cre;TGwt^ samples. All used animals for western analysis were 8 months of age. (C) Densitometric quantification of NMDA receptor subunits of three different experiments using different animals. The respective subunit was normalized to GAPDH. (D) Representative western blot of AMPA receptor subunits 2/3/4 and PSD‐95 for normalization. The dotted red line separates mepβ^Cre;wt/wt^ from mepβ^Cre;TG/wt^ animals. All animals used for western analysis were 8 months of age. (E) Densitometric quantification of AMPA receptor subunits of three different experiments using different animals normalized to PSD‐95.

For a more accurate assessment of these receptors, we isolated and enriched synaptic proteins. This process involved dissecting the hippocampus and cortex and performing gradient centrifugation, resulting in the isolation of the cytosolic (S2) and synaptosomal fractions (Figure 5A). We focused on the synaptosomal fraction due to the enriched membrane‐bound synaptic proteins (Figure S2). Western blot analysis revealed no significant differences in the expression levels of NMDA receptor subunits 1A, 2A, or 2B within this fraction (Figure 5B,C).

After examining the NMDA receptor subunits, we shifted our focus to the AMPA receptor, as it also plays an important role in learning and memory [24]. Similar to the NMDA receptor, there was no difference in the expression of the AMPA receptor between mepβ^Cre;wt/wt^ mice and meprin β‐overexpressing animals. A quantification across three independent experiments, each with distinct animal samples, did not reveal any statistically significant changes (Figure 5D,E).

To analyze a potential processing effect of meprin β on NMDA‐receptor subunits, we performed cotransfection experiments in HEK293T cells. Similar to the western analysis results, we did not see any changes in NMDA‐receptor subunit expression, regardless of the overexpression of meprin β (Figure S3).

These data indicate that the behavioral and electrophysiological effects of meprin β are not likely mediated by changes in NMDA or AMPA receptor expression. Instead, perhaps a more subtle synaptic alteration is responsible for the observed phenotype.

### 3.6 Utilization of N‐Terminomics for the Identification of Novel Substrates of Meprin β in the Murine Brain

**FIGURE 6:**
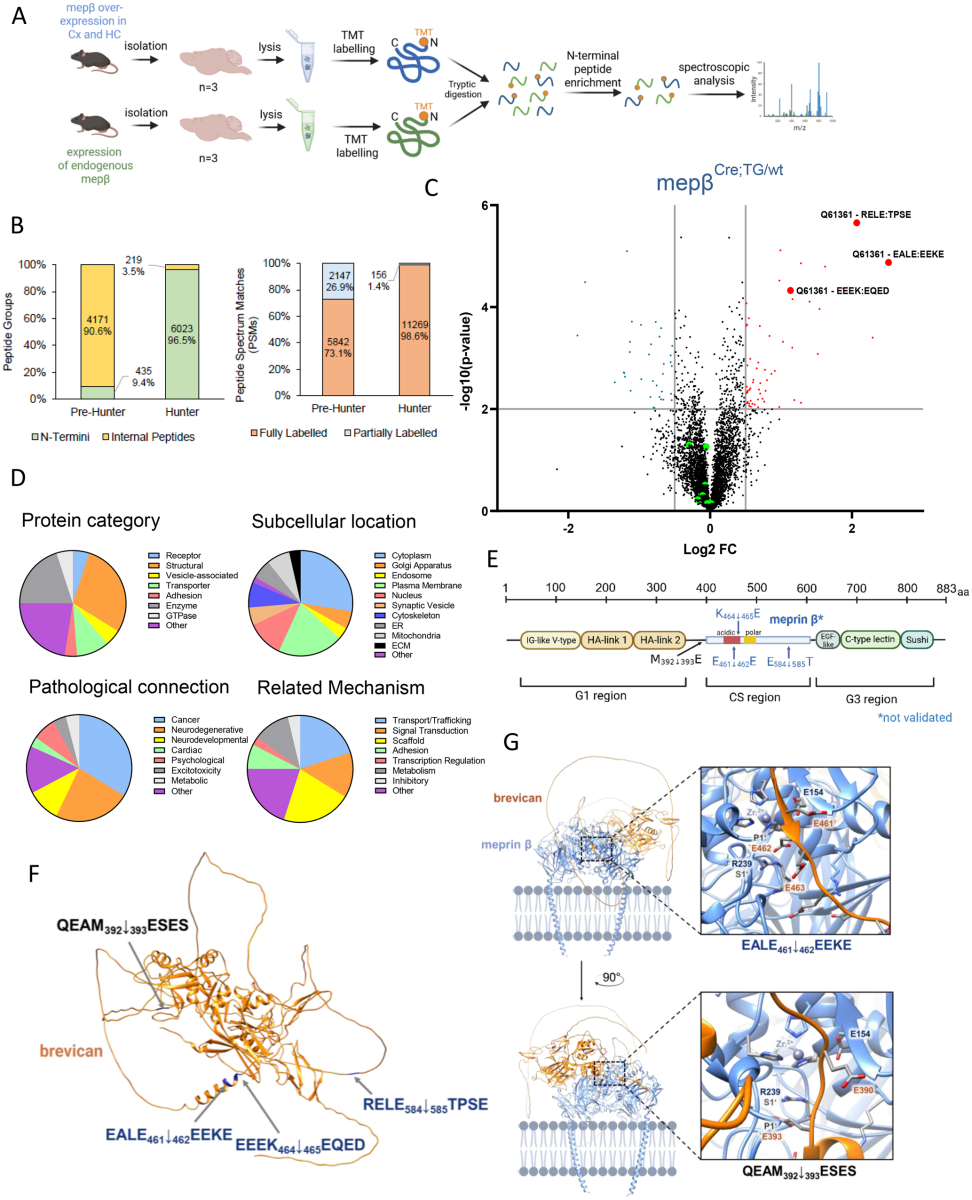
Utilization of N‐terminomics for the identification of novel substrates of meprin β within the brain. (A) Schematic overview of the N‐terminomics process: N‐terminal peptides were enriched using the TMT HUNTER protocol before spectroscopic analysis. Brains of three animals were collected and used per group. (B) Quantification of enriched peptides pre‐ and post‐HUNTER. (C) Volcano plot visualization of potential substrates: Red dots show the overrepresented peptides in meprin β‐overexpressing brains, and the blue dots show the underrepresented peptides. The threshold was set at log_2_ (difference) = ±0.5 and –log_10_ (*p* value) = 2. Additionally, depicted are the P4–P4′ amino acid residues surrounding the N‐terminal cleavage site of the respective brevican peptide. The green dots in the center represent the detected peptides of NMDA and AMPA receptor subunits, confirming the absence of overrepresentation, consistent with the results from western blotting experiments. (D) Categorization of obtained peptides in different functional areas for visual overview. (E) Schematic overview of brevican and potential cleavage sites indicated by blue arrows as predicted by N‐terminomics analysis. With a black arrow an additional potential cleavage site by meprin β based on alpha‐fold modeling (G) is shown. (F) Predicted protein structure of brevican generated with AlphaFold 3 [25] using the UniProt Consortium 2021 sequence (uniprot ID: Q61361) and illustrated with UCSF Chimera. Cleavage sites identified for meprin β by N‐terminomics are labeled in blue, additional potential cleavage site is labeled in black. (G) Predicted structure of brevican (uniprot ID: Q61361) together with meprin β homodimer (uniprot ID: Q61361) generated with AlphaFold 3 [25] using the UniProt Consortium 2021 sequence and illustrated with UCSF Chimera. The cleavage site E461↓E462 within brevican identified by N‐terminomics for meprin β nicely fits into the first active site of the meprin β homodimer. The second active site aligns with the sequence QEAM392↓393ESES, suggesting a potential additional cleavage by meprin β at this site.

Our investigations into meprin β overexpression in cortical and hippocampal neurons led us to hypothesize that additional substrates for meprin β may exist in the brain influencing learning and memory. To address mechanisms affected by meprin β processing and identify additional substrates, we employed N‐terminomics using HUNTER analysis on brain lysates from meprin β overexpressing and respective Cre‐negative control animals (Figure 6A). A key benefit of this method is that it enables the enrichment of N‐terminally truncated peptides from minimal sample quantities, allowing for a robust, sensitive, and scalable analysis [22]. The HUNTER approach identified substantially more peptides than pre‐HUNTER analysis, demonstrating the effectiveness of N‐terminal peptide enrichment prior to MS analysis (Figure 6B). Using this approach, we discovered a set of N‐terminal peptides (Figure S4) that are significantly increased in meprin β‐overexpressing animals (Figure 6C—red dots). Criteria for significant overrepresentation were defined as −log_10_ (*p* value) = 3 and log_2_ (expression difference) = ±0.5 (Figure 6C).

We observed particularly high enrichment values for brevican (Q61361), aligning with observations in astrocytes reported by Armbrust et al. [18]. Additionally, the cleavage site of the discovered overrepresented brevican peptides aligns well to the established meprin β cleavage motif [12, 26] as meprin β prefers negatively charged residues, such as aspartate (D) and glutamate (E), at the P1 or P1’ positions. Given brevican's established involvement in LTP [27] and brain ECM [28], this protein emerges as a potential proteolytic substrate for meprin β, supporting the observed behavioral and electrophysiological results.

Following the categorization of overrepresented peptides in meprin β‐overexpressing animals, we observed a marked increase in potential substrates associated with the scaffolding of the cell (Figure 6D, “protein category”—orange), the plasma membrane (Figure 6D, “subcellular location”—green), influence in neurological pathologies (Figure 6D, “pathological connection”—orange/yellow/red), and the scaffolding mechanism (Figure 6D, “mechanism”—yellow).

Additionally, NMDA‐ and AMPA‐receptor subunits showed no significant overrepresentation nor underrepresentation in meprin β‐overexpressing mice, further suggesting that both receptors play no substantial role in meprin β processing as indicated by western blot analysis and cotransfection experiments (Figure 6C—green dots and Figure S3).

N‐terminomics analysis revealed multiple brevican peptides overrepresented in meprin β‐overexpressing animals. Brevican is a dumbbell‐shaped molecule comprising N‐terminal (G1) and C‐terminal (G3) domains, separated by a polar, negatively charged central spacer (Figure 6E). This spacer contains chondroitin sulfate (CS) attachment sites and meprin β cleavage sites identified by N‐terminomics (indicated by blue arrows) (Figure 6E,F).

Utilizing AlphaFold 3 (PMID: 38718835) and the UniProt Consortium 2021 sequence, we modeled brevican (UniProt ID: Q61361) with a meprin β homodimer (UniProt ID: Q61361) (Figure 6G). The cleavage site E461↓E462 within brevican, identified by N‐terminomics for meprin β, aligns with the first active site of the meprin β homodimer, supporting our experimental N‐terminomics data. The additional cleavage site at K464↓E465, in close proximity, suggests that meprin β may shift the cleavage site by three amino acids or that cleavage at K464↓E465 occurs following initial cleavage at E461↓E462. The second active site aligns with the sequence QEAM392↓393ESES, indicating a potential additional cleavage by meprin β at this site. The absence of this cleavage site in the N‐terminomics data could be due to methodological limitations or further downstream processing of brevican cleavage products by other proteases like ADAMTS4. Additionally, N‐terminomics identified cleavage at E584↓T585. Although our AlphaFold modeling lacks validation for this site, it is considered probable, as it aligns with the cleavage preference of meprin β [26] and is likely accessible based on the predicted structure. However, our AlphaFold modeling does not account for glycosylation, which may impact accessibility and proteolytic processing by meprin β.

### 3.7 Meprin β Overexpression Causes Cleavage of Full‐Length Brevican

**FIGURE 7:**
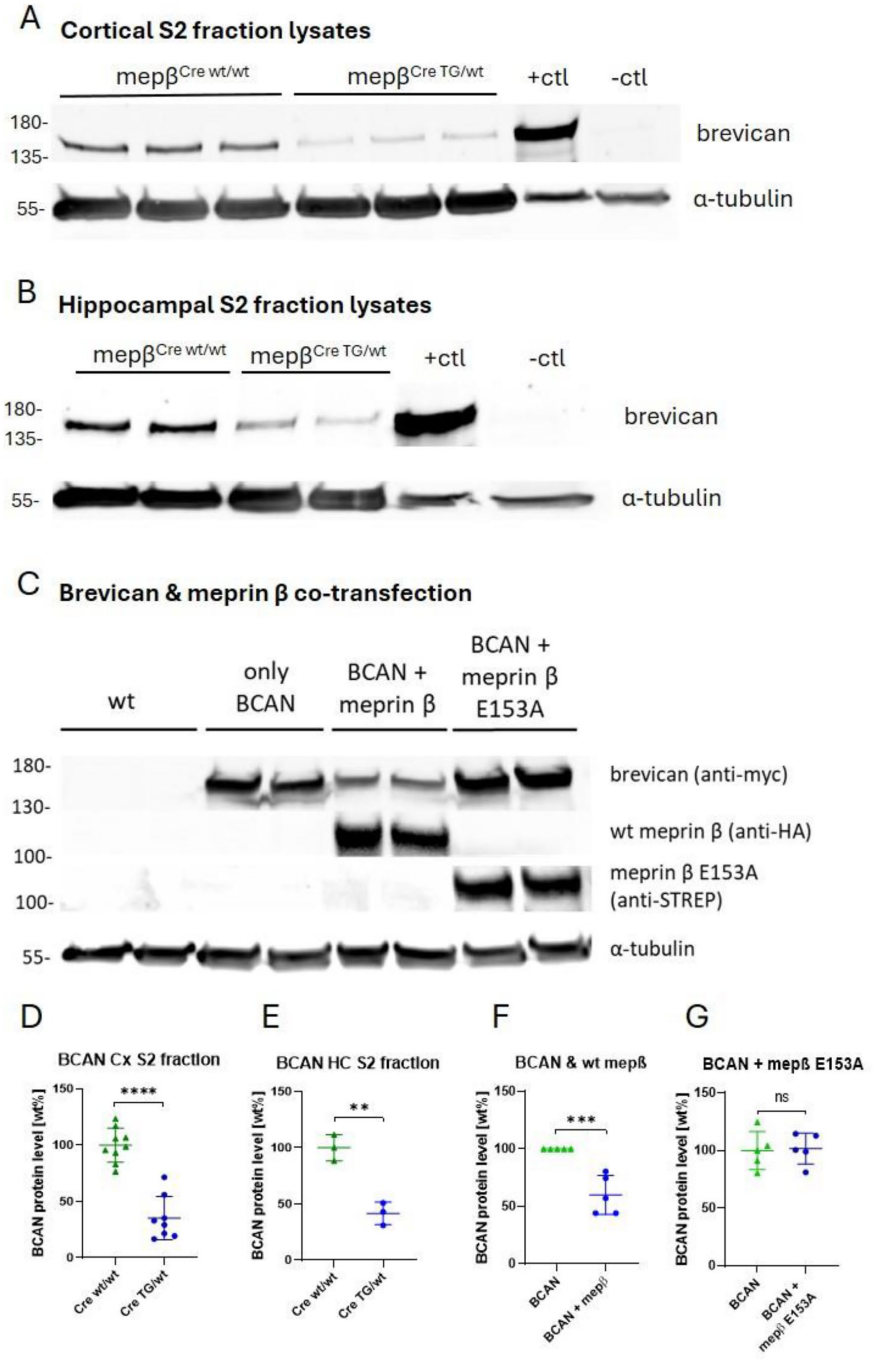
Meprin β overexpression causes cleavage of ECM protein brevican in mouse cortex, hippocampus and transiently cotransfected HEK 293T cells. Brains from animals used in this analysis were 8 months old. (A, D) Western blot analysis examined the soluble synaptosome fraction (S2) from cortices of mepβ^Cre;wt/wt^ and mepβ^Cre;TG/wt^ mice, with meprin β KO‐HEK293T cells transfected with brevican serving as a positive control and kidney lysate as a negative control. Samples were analyzed across three independent experiments, with full‐length brevican levels normalized to tubulin. Statistical analysis (Student's *t*‐test) revealed a significant reduction in full‐length brevican in the cortical S2 fraction of meprin β‐overexpressing mice. (B, E) Western blot analysis of brevican and tubulin was conducted on S2 fractions from hippocampi of mepβ^Cre;wt/wt^ and transgenic mice. Due to low sample yields, fractions were pooled from 2 to 3 animals per genotype. Data points represent pooled samples analyzed across three independent experiments. Densitometric quantification revealed a significant reduction in full‐length brevican in the hippocampal S2 fractions of meprin β‐overexpressing mice, normalized against tubulin. Statistical analysis (Student's *t*‐test) revealed a significant reduction in full‐length brevican in the hippocampal S2 fraction of meprin β‐overexpressing mice. (C, F, G) Western blot analysis was applied to investigate protein interactions in HEK293T cells cotransfected with brevican and either wt meprin β or the catalytically inactive meprin β variant E153A. Overexpression of tagged meprin β and E153A was confirmed with HA‐/STREP‐tag antibodies. From left to right the experimental setup included wild‐type HEK293T cells as a negative control, single transfections and cotransfections. Results showed that cotransfection of brevican with wt meprin β significantly reduced the level of full‐length brevican, whereas cotransfection with the inactive E153A variant did not affect brevican levels, suggesting that the proteolytic activity of meprin β is necessary for brevican cleavage.

Brevican is long known to affect synaptic plasticity [27]. Its appearance in the N‐terminomics analysis of potential meprin β substrates therefore offers an explanation for the phenotype seen in transgenic meprin β‐overexpressing mice. Brevican was detected in the “Soluble fraction” (S2) of synaptosome isolation from mouse brains in both mepβ^Cre;wt/wt^ mice and transgenic mice overexpressing meprin β. However, SDS‐PAGE and western blot analyses revealed significantly less full‐length brevican in cortices and hippocampi of mepβ^Cre;TG/wt^ transgenic mice, suggesting brevican as a potential meprin β target in vivo in the mouse brain (Figure 7A,B,D,E).

To further confirm and validate meprin β influence on brevican expression, myc‐tagged brevican was transiently cotransfected in HEK 293T cells with meprin β. SDS Page and western blotting of cell lysates revealed a significant decrease in full‐length brevican in the cotransfected lysates with wt meprin β (Figure 7C,F) confirming once more its influence on brevican.

To test whether the reduction in brevican expression upon wt meprin β overexpression is due to proteolytic activity, the cells were cotransfected with a proteolytically inactive variant of meprin β (E153A). No reduction in brevican protein level was observed in the presence of the inactive meprin β variant, confirming that the decrease in brevican protein levels is directly linked to the proteolytic activity of meprin β (Figure 7C,G).

### 3.8 Meprin β‐Overexpressing Mice Display a Higher Hippocampal Network Excitability Than Mepβ^Cre^ ^;wt/wt^ Mice

As changes in the brain ECM have been documented to influence AMPAR‐dependent excitability, we next investigated whether an overexpression of meprin β in the mouse hippocampus affects the AMPAR‐dependent excitability of the hippocampal neuronal network [27, 28]. We performed electrophysiological recordings of acute hippocampal slices by use of the MEA in the mepβ^Cre;TG/wt^ and mepβ^Cre;wt/wt^ mice. A series of electrical stimulations ranging from 0.5 V to 5 V, with step increases of 0.5 V, was applied to one electrode of the MEA located below the Schaffer collaterals in the CA3 region of the brain slice. The resulting extracellular fEPSPs were recorded with the MEA electrode below the target region in CA1. The amplitude of these fEPSP signals allowed us to determine the excitability in the CA3 to CA1 signaling pathway.

**FIGURE 8:**
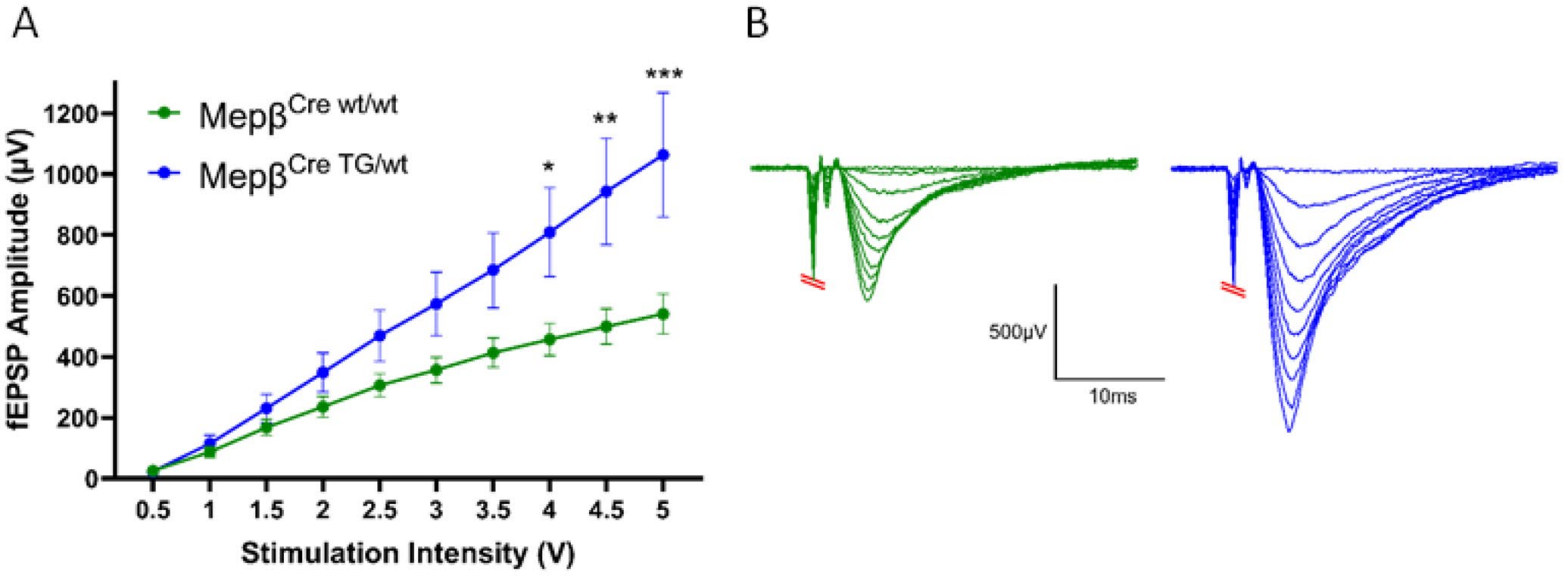
Meprin β‐overexpressing mice display a significantly higher evoked network activity than mepβ^Cre;wt/wt^ mice. (A) Multi‐electrode array (MEA) recordings displaying Input/Output (I/O) curves with the stimulation input ranging from 0.5 V to 5 V in 0.5 V steps. The output was recorded as field excitatory postsynaptic potentials (fEPSPs). Statistical analysis using two‐way ANOVA with post hoc Šídák's multiple comparisons test. Graph shows mean ± SEM. Mepβ^Cre;wt/wt^ *n* = 10 slices from four mice. mepβ^Cre;TG/wt^ *n* = 9 slices from 4 mice. *p* = 0.05. (B) Representative fEPSP traces were recorded from the gradually increasing stimulus intensity from the I/O curve for mepβ^Cre;wt/wt^ (green) and mepβ^Cre;TG/wt^ (blue) mice.

Interestingly, the amplitude of the fEPSPs obtained from the mepβ^Cre;TG/wt^ mice was significantly larger than those from mepβ^Cre;wt/wt^ mice at stimulation intensities from 4 V through to 5 V (Figure 8A). Representative fEPSP traces with overlaid outputs from the input/output curve can be seen in Figure 8B.

While the amplitude of such I/O‐curves is generally dominated by activity from postsynaptic excitatory AMPARs, we wanted to disclose whether or not presynaptic mechanisms were involved in the observed increase of evoked fEPSP amplitudes. To investigate this, we carried out paired‐pulse stimulations on MEA‐electrodes located in area CA3 with an inter‐stimulus interval (ISI) of 50 ms. However, there were no differences in PPR found between mepβ^Cre;wt/wt^ and mepβ^Cre;TG/wt^ mice (Figure S8).

We concluded that overexpression of meprin β in the mouse hippocampus significantly increased neuronal network excitability between CA3 and CA1, and these changes are likely due to changes in brevican expression and alterations at the postsynapse.

## 4 Discussion

Brevican is expressed in both neurons and glial cells, where it is predominantly localized at perisynaptic sites. Together with other lecticans, hyaluronic acid, and tenascin‐R, brevican plays a crucial role in the formation of perineuronal nets (PNNs). These nets are structures that surround the proximal dendrites and neuronal cell bodies, stabilizing synapses by acting as a barrier against the growth of nearby axons or dendrites [29, 30, 31]. Moreover, PNNs are essential for long‐term memory consolidation [32], extending the importance of brevican in the neuronal ECM and its role in synaptic transmission to behavioral and cognitive aspects. Brevican is a key contributor to spatial memory and memory retrieval function, as found previously through hippocampal proteoglycan isolation and characterization in rats that underwent the Morris' Water Maze spatial paradigm test [32]. Brevican‐deficient mice have also displayed impaired hippocampal CA1 LTP in previous studies, with LTP considered the most widely established underlying cellular mechanism for learning and memory consolidation [27]. Even within clinical cohorts, patients suffering from vascular dementia were found to have significantly decreased serum brevican levels compared to that of healthy controls [33, 34, 35, 36, 37, 38], which prompted the suggestion of the use of fluid brevican as a biomarker for dementia‐ and cognitive impairment‐related brain pathologies. In a recent study by Liu et al. [39], brevican was further implicated in ACD, Alzheimer's disease (AD), stroke, and depression, underscoring its potential utility as a biomarker for a more diverse array of diseases. Notably, Liu et al. demonstrated that brevican exhibited no influence on movement, a finding that aligns with our observations (Figure S5).

To better understand how meprin β overexpression influences synaptic function, we examined its subcellular localization using confocal microscopy on sagittal brain slices, specifically targeting the CA3 region of the hippocampus. This region was selected due to previously observed deficits in spatial learning in the Morris' water maze in meprin β‐overexpressing mice. Wild‐type animals showed meprin β predominantly localized around the nucleus, likely reflecting the endoplasmic reticulum. In contrast, overexpressing mice exhibited strong meprin β expression in the soma, axons, and notably at synaptic sites. Costaining with the postsynaptic marker PSD‐95 revealed a higher degree of overlap compared to the presynaptic marker VGLUT, supporting postsynaptic enrichment of meprin β. This subcellular distribution provides a potential anatomical correlate to the observed electrophysiological and behavioral changes.

While LTP induction is primarily an NMDA receptor (NMDAR)‐dependent mechanism, brevican has also been highlighted for its associations with AMPA receptors (AMPAR); namely their mobility and diffusion [33, 40] and the capacity at which they can facilitate short‐term synaptic plasticity [41, 42]. Knockout of AMPAR subunit GluR1 in mice exhibits impaired learning and memory, which also highlights AMPA's role in synaptic plasticity and associative learning [43, 44].

On discovering that overexpression of meprin β resulted in the cleavage of such a main component of the neuronal ECM as brevican, the electrophysiology and behavioral results also became more coherent. The increase in basal excitability, seen by the significantly larger evoked input/output curve amplitudes in meprin β‐overexpressing mice compared to mepβ^Cre;wt/wt^ mice, is likely due to the increased AMPAR mobility and lateral diffusion in the postsynaptic CA1 pyramidal cells [40]. Interestingly, it was shown that the surface compartments formed by the ECM—of which brevican is a main component—hinder lateral diffusion of AMPA receptors and may therefore decrease the capacity for short‐term synaptic plasticity [40]. With the digestion of brevican and therefore an at least partial breakdown of the impeding ECM, AMPARs, which are desensitized from use, can quickly be replaced by naïve AMPARs moving from the extrasynaptic space to the postsynaptic density (PSD), ready for activation. This means that the number of postsynaptic receptors that are available for activation can be consistently replenished, enhancing the AMPAR‐dependent evoked activity responses seen from the input/output curve. A study by Favuzzi et al. [45] supports previous findings by demonstrating that the PNN surrounding parvalbumin (PV+) interneurons plays a critical role in regulating plasticity. The authors propose that brevican, a key component of the PNN, modulates cellular and synaptic plasticity by influencing the localization of potassium channels and AMPA receptors [45].

The fact that meprin β cleaves a protein of the ECM within which the receptors are immobilized—allowing a more fluid diffusion of synaptic receptors in the membrane, rather than cleaving or affecting those receptors directly—brings with it some further inquiries. This assumes that total AMPAR and/or NMDAR subunit protein expression levels in the meprin β‐overexpressing mouseline should not be different from that of the mepβ^Cre;wt/wt^ controls, despite electrophysiological changes which are normally receptor concentration‐dependent. Indeed, quantitative western blot analysis confirmed that total expression levels for AMPAR and NMDAR subunits were equivalent for both animal groups (Figure 5), owing to the point that it is not about whether the receptors (or their subunits) are increased or decreased in expression, but rather where they are in the membrane/ECM at any given moment. If meprin β was in fact actively degrading both NMDA and AMPA receptors, it would likely result in early mortality, given their developmental roles [46]. Even if mortality did not occur, it would likely cause a distinct phenotype in the transgenic animals, since the meprin β overexpression is occurring from the beginning. Meprin β‐overexpressing mice, however, exhibit no developmental abnormalities and appear phenotypically normal (own observations). The absence of a clear phenotype of meprin β‐overexpressing mice might relate to the fact that the natural increase of brevican expression only normally occurs *after* birth [47]. Supposedly, brevican is disposable in early development and not essential for fetal growth or embryonic development.

Additionally, the significant intergroup electrophysiological changes could be specifically due to receptor expression on the membrane surface, rather than to total expression. This was also thoroughly considered. Since there was a significant increase in evoked fEPSPs concomitantly with a significant decrease/loss of LTP in the meprin β‐overexpressing mice, a metaplasticity effect was hypothesized. In order to investigate this, a surface biotinylation assay on primary hippocampal neurons from mepβ^Cre;wt/wt^ and meprin β KO mouse brains was undertaken (Figure S7). This assay showed no changes in membrane surface AMPAR subunit or NMDAR subunit expression, verifying that membrane surface receptor composition was not quantitatively altered between these groups, as well as implying that meprin β does not influence membrane surface receptor expression. Furthermore, we would like to emphasize that Brakebusch et al. [27] reported an impairment in LTP maintenance in brevican KO mice, which was attributed to structural and functional changes in the ECM affecting synaptic signaling and maintenance. This finding supports our theory, since a similar effect is observed in meprin β‐overexpressing mice.

While the evoked network excitability and the LTP experiments proved significant differences between groups, the additional electrophysiological paired‐pulse ratio (PPR) experiments did not (Figure S8). Although an increase in synaptic facilitation is generally seen with an accompanying increase in PPR, the significant increase of both the first and the second evoked response from the PPR protocol in these meprin β‐overexpressing mice meant that the overall ratio of the second response relative to the first was equivalent across groups. This is, however, in line with our hypothesis that the postsynaptic replenishment of naive AMPARs causes the significant enhancement of the evoked response amplitudes—something which does not concern the presynaptic release probability (which is reflected by the PPR) but rather the postsynaptic AMPAR functional efficiency.

Of course, meprin β overexpression could also owe to morphological changes in neurons or their connectivity. The composition of extracellular matrix (ECM) proteins critically impacts neuronal spine density and synaptic plasticity, which are vital for learning and memory [21]. Enzymes like MMP‐9 cleave ECM components, enhancing dendritic spine formation and stability [48]. Such modifications may, of course, contribute to impairments in synaptic function and cognitive processes in the brain, showing the large impact proteolytic enzymes have on the ECM. For example, ADAMTS4—a brevican‐cleaving proteolytic enzyme—strongly influences neuronal spine density and is essential for synaptic plasticity [49, 50] as well as being linked to LTP [27]. Our data strongly reflect these changes: meprin β overexpression leads to lower brevican protein levels and ECM proteolysis, which then influences LTP formation and ultimately impacts learning and memory.

In conclusion, brevican emerges as a pivotal ECM component integral to synaptic plasticity, memory formation, and cognitive function. The interplay between brevican and meprin β highlights the dynamic regulation of synaptic receptors and neuronal connectivity, emphasizing how ECM proteolysis impacts neuronal signaling and plasticity. These findings not only underscore meprin β's role in memory and synaptic transmission but also put forward its potential as a biomarker for cognitive disorders.

## Author Contributions

C.U.P. designed the study. L.M. and M.K. generated and maintained the novel mouse line. M.K. and U.S. performed behavioral experiments. C.G. conducted the electrophysiological experiments. S.K. and D.T. performed western blotting experiments, and M.K. and C.G. performed biotinylation analysis. N‐terminomics was performed by A.T. and M.A., while data were analyzed by A.T., M.A., and M.K. K.B. generated Meprin β KO cells. M.K., C.G., and S.K. wrote the draft, which was supervised and edited by C.U.P., T.M., C.B.‐P., and K.B.

## Ethics Statement

All animal studies were conducted in compliance with European and German guidelines for the care and use of laboratory animals and were approved by the Central Animal Facility of the University of Mainz and the ethical committee on animal care and use of Rhineland–Palatinate, Germany.

## Consent

All authors read and approved the final manuscript.

## Conflicts of Interest

The authors declare no conflicts of interest.

## Supporting information


Figures S1–S8.

## Data Availability

All primary data and materials in the manuscript are available upon reasonable request.
