# Supplementary material for: Meprin β Modulates Brevican Proteolysis Impairing Neural Plasticity and Memory Formation
Source: FASEB J. 2025 May 21;39(10):e70616. doi: 10.1096/fj.202500017R (PMC12093287; doi:10.1096/fj.202500017R)
Supplement: Supplementary file 1 — Figures S1–S8. [file FSB2-39-e70616-s001.pdf]

Supplementary data

Meprin β Modulates Brevican Expression Impairing Neural Plasticity and Memory Formation

Immunofluorescence staining of brain slices from mepβ$^{Cre;wt/wt}$ and mepβ$^{Cre;TG/wt}$ animals using confocal microscopy

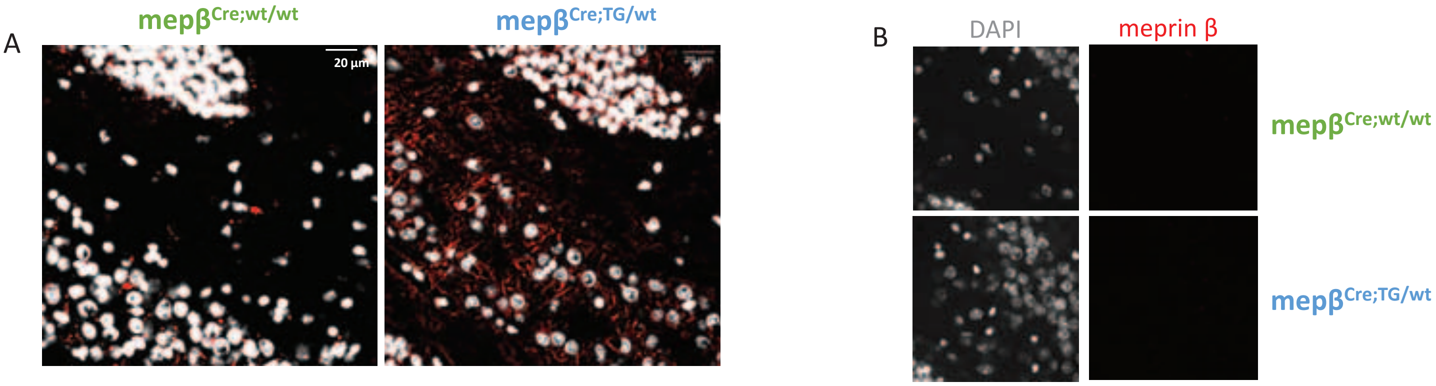

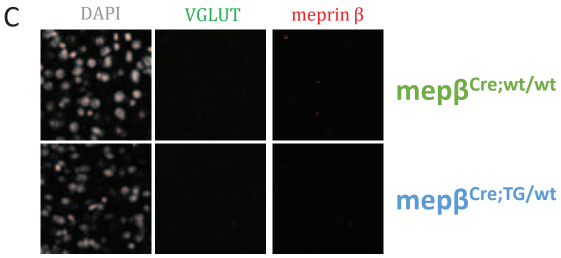

Suppl. Figure 1. (A) Immunofluorescent staining of HA-tagged transgenic meprin β in mepβ$^{Cre;wt/wt}$ and mepβ$^{Cre;TG/wt}$ animals with an anti-HA tag antibody to visualise the overexpression of meprin β. Pseudo-colors: Grey (DAPI, 1:200), red (mouse anti-HA, 2-2.2.14 Invitrogen, 1:100; donkey anti-mouse IgG-IRDye680RD, Li-Cor Biosciences, 1:200). Red staining in left image is unspecific background. **(B)** Control stainings that were incubated only with the secondary antibody IRDye680RD (right) (925-68074, Li-Cor Biosciences, 1:200, rabbit). **(C)** Control stainings that were incubated only with the secondary antibody. PSD95 is absent, because it was stained only with a fluorophore-attached primary antibody. Pseudo-colors: Grey (DAPI), green (rabbit anti-VGLUT, 1:40, donkey anti-rabbit-AF488, 711-545-152 Jackson Lab. Biozol, 1:200), red (goat anti-meprin β, PA5-47474; ThermoFisher Scientific, 1:40; donkey anti-goat IgG-IRDye680RD, 925-68074, Li-Cor Biosciences, 1:200).

Synaptosome Isolation of Cortex and Hippocampus

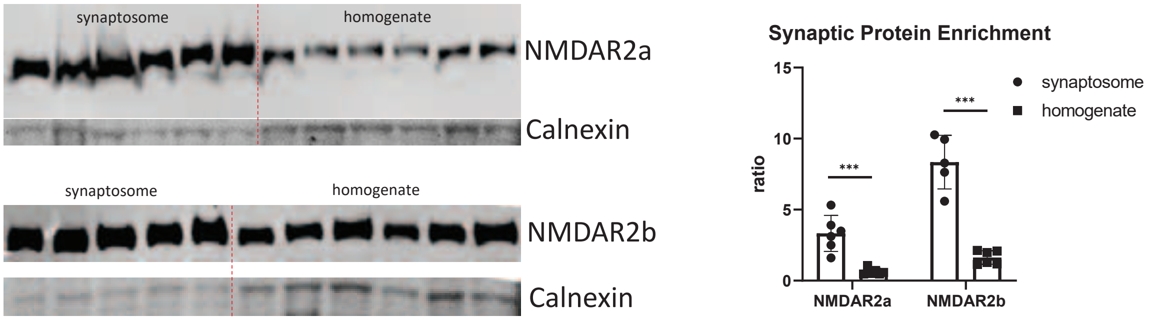

Suppl. Figure 2. Enrichment of synaptic proteins. Brains of mice were dissected into cortex and hippocampus and homogenized. To address synaptic proteins more clearly, synaptic proteins were enriched using the Syn-PER extractions reagent as recommended by the manufacturer. Western blotting confirmed an enrichment of synaptic proteins (here representatively shown are NMDAR2a and NMDAR2b receptor subunits). Also densitometric analysis revealed a clear enrichement after isolation with the Syn-PER reagent.

Co-Transfection of Meprin β and NMDA receptor subunits in HEK293T cells

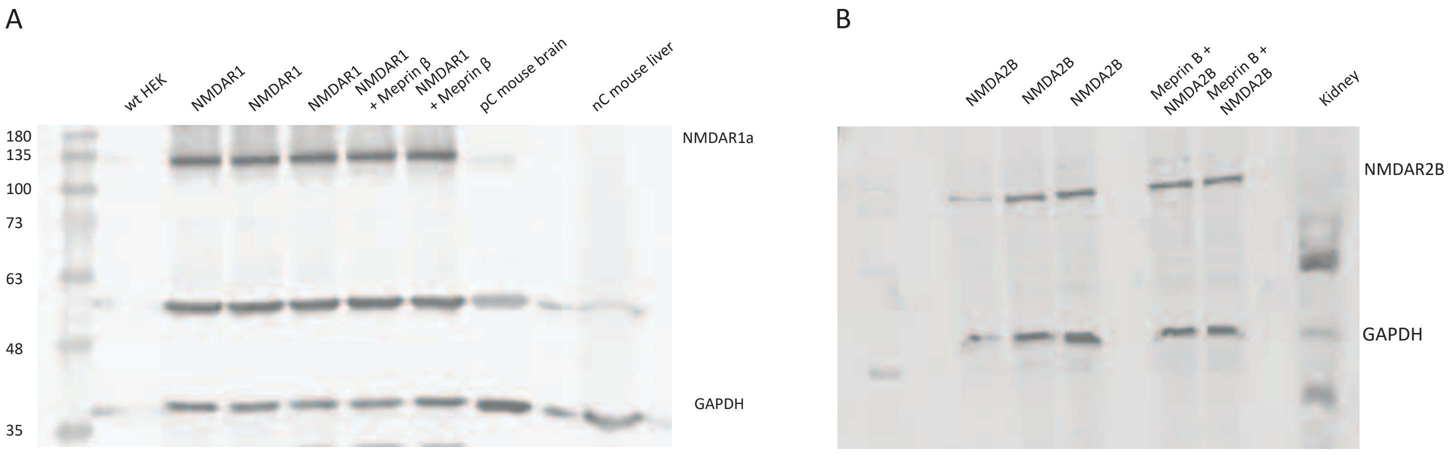

Suppl. Figure 3. Transfection of NMDA subunits. Co-Transfection of NMDAR2a or NMDAR2b with meprin β in HEK293T cells revealed no differences in protein expression between meprin β-overexpressing and wt animals. That observation fits to the western blotting results of the brain lysated.

Overrepresented Peptides in Meprin β-overexpressing Animals

A

	Master Protein Accession	Master Protein Description	Peptide sequence	Log2 FC
3	Q61361	Brevican core protein OS=Mus musculus OX=10090 GN=Bcan PE=1 SV=2	TPSEEKSGR	2.516
4	Q61361	Brevican core protein OS=Mus musculus OX=10090 GN=Bcan PE=1 SV=2	EEKEQEDLWVWPR	2.069
5	P0C192	Leucine-rich repeat-containing protein 4B OS=Mus musculus OX=10090 GN=Lrrc4b PE=1 SV=1	TQPGEEAQQPR	1.622
6	P55066	Neurocan core protein OS=Mus musculus OX=10090 GN=Ncan PE=1 SV=1	TTATEKGLR	1.163
7	Q8C419	Probable G-protein coupled receptor 158 OS=Mus musculus OX=10090 GN=Gpr158 PE=1 SV=2	DPPGRPDSPR	1.403
8	P55066	Neurocan core protein OS=Mus musculus OX=10090 GN=Ncan PE=1 SV=1	DTTATEKGLR	1.279
9	Q9QUP5	Hyaluronan and proteoglycan link protein 1 OS=Mus musculus OX=10090 GN=Hapln1 PE=1 SV=1	DSYTPPDQDR	1.282
10	B9EKR1	Receptor-type tyrosine-protein phosphatase zeta OS=Mus musculus OX=10090 GN=Ptprz1 PE=1 SV=1	EEYGKDNEEDTGLNPGR	0.977
11	Q06890	Clusterin OS=Mus musculus OX=10090 GN=Clu PE=1 SV=1	DNELQELSTQGSR	1.027
12	Q80TS3	Adhesion G protein-coupled receptor L3 OS=Mus musculus OX=10090 GN=Adgrl3 PE=1 SV=3	SELERPPVR	1.159
13	Q9QUP5	Hyaluronan and proteoglycan link protein 1 OS=Mus musculus OX=10090 GN=Hapln1 PE=1 SV=1	SYTPPDQDR	0.775
14	Q8CAE9	Podocalyxin-like protein 2 OS=Mus musculus OX=10090 GN=Podxl2 PE=1 SV=2	EEEEEEEEEEEEREEEER	0.707
15	P55066	Neurocan core protein OS=Mus musculus OX=10090 GN=Ncan PE=1 SV=1	DTQDTTATEKGLR	0.864
16	Q4KMM3	Oxidation resistance protein 1 OS=Mus musculus OX=10090 GN=Oxr1 PE=1 SV=3	EEQNNSANTQKHPSR	0.551
17	Q61361	Brevican core protein OS=Mus musculus OX=10090 GN=Bcan PE=1 SV=2	EQEDLWVWPR	1.134
18	P20357	Microtubule-associated protein 2 OS=Mus musculus OX=10090 GN=Map2 PE=1 SV=2	SENLSGESGSFYEGTDDKVR	0.689
19	O88935	Synapsin-1 OS=Mus musculus OX=10090 GN=Syn1 PE=1 SV=2	SPGATPGSATASAER	0.680
20	P43006	Excitatory amino acid transporter 2 OS=Mus musculus OX=10090 GN=Slc1a2 PE=1 SV=1	HDSHLSSDEPKHR	1.188
21	Q9WV92	Band 4.1-like protein 3 OS=Mus musculus OX=10090 GN=Epb41l3 PE=1 SV=1	HSTPVKR	0.654
22	P43006	Excitatory amino acid transporter 2 OS=Mus musculus OX=10090 GN=Slc1a2 PE=1 SV=1	HLSSDEPKHR	0.619
23	O08539	Myc box-dependent-interacting protein 1 OS=Mus musculus OX=10090 GN=Bin1 PE=1 SV=1	AQPSDNAPEKGNKSPSPPPDGSPAATPEIR	0.526
24	P01643	Ig kappa chain V-V region MOPC 173 OS=Mus musculus OX=10090 PE=1 SV=1	DIQMTQTTSSLSASLGDR	0.578
25	Q8CI94	Glycogen phosphorylase, brain form OS=Mus musculus OX=10090 GN=Pygb PE=1 SV=3	IKKDPAKAFVPR	0.546
26	Q9JIF7	Coatomer subunit beta OS=Mus musculus OX=10090 GN=Copb1 PE=1 SV=1	KEHPAHER	0.768
27	P50544	Very long-chain specific acyl-CoA dehydrogenase, mitochondrial OS=Mus musculus OX=10090 GN=Acadvl PE=1 SV=3	DKPETLSSDASTR	0.706
28	P68368	Tubulin alpha-4A chain OS=Mus musculus OX=10090 GN=Tuba4a PE=1 SV=1	GDVVPKDVNAAIAAIKTKR	0.546
29	P19783	Cytochrome c oxidase subunit 4 isoform 1, mitochondrial OS=Mus musculus OX=10090 GN=Cox4i1 PE=1 SV=2	SVVKSEDYAFPTYADR	0.544
30	Q9CY16	28S ribosomal protein S28, mitochondrial OS=Mus musculus OX=10090 GN=Mrps28 PE=1 SV=1	STESGSESATHDSSAPR	0.538
31	Q8BGY7	Protein FAM210A OS=Mus musculus OX=10090 GN=Fam210a PE=1 SV=1	STSQETPSEKKEETDPLQDKSISLYQR	0.824
32	Q03265	ATP synthase subunit alpha, mitochondrial OS=Mus musculus OX=10090 GN=Atp5f1a PE=1 SV=1	TSVDLEETGR	0.679
33	P97807	Fumarate hydratase, mitochondrial OS=Mus musculus OX=10090 GN=Fh PE=1 SV=3	FDTFGELKVPTDKYYGAQTVR	0.596
34	P14231	Sodium/potassium-transporting ATPase subunit beta-2 OS=Mus musculus OX=10090 GN=Atp1b2 PE=1 SV=2	VSDHTPKYQDR	0.785
35	P14873	Microtubule-associated protein 1B OS=Mus musculus OX=10090 GN=Map1b PE=1 SV=2	IAASGPVKELEAER	0.556
36	Q9ESM3	Hyaluronan and proteoglycan link protein 2 OS=Mus musculus OX=10090 GN=Hapln2 PE=2 SV=1	HYLLPPIHEVIHSR	0.526
37	P56399	Ubiquitin carboxyl-terminal hydrolase 5 OS=Mus musculus OX=10090 GN=Usp5 PE=1 SV=1	FSIPDFQR	0.625
38	Q8K1Z0	Ubiquinone biosynthesis protein COQ9, mitochondrial OS=Mus musculus OX=10090 GN=Coq9 PE=1 SV=1	SETQGPEFSRPPPR	0.552
39	P39053	Dynamin-1 OS=Mus musculus OX=10090 GN=Dnm1 PE=1 SV=2	VSPANSDLANSDALKIAKEVDPQGQR	0.630
40	P50544	Very long-chain specific acyl-CoA dehydrogenase, mitochondrial OS=Mus musculus OX=10090 GN=Acadvl PE=1 SV=3	AVLDKPETLSSDASTR	0.605
41	P54071	Isocitrate dehydrogenase [NADP], mitochondrial OS=Mus musculus OX=10090 GN=Idh2 PE=1 SV=3	VEKPVVEMDGDEMTR	0.514
42	P63158	High mobility group protein B1 OS=Mus musculus OX=10090 GN=Hmgb1 PE=1 SV=2	GKGDPKKPR	0.701
43	Q04447	Creatine kinase B-type OS=Mus musculus OX=10090 GN=Ckb PE=1 SV=1	NSHNTQKLR	0.569
44	Q9Z0E0	Neurochondrin OS=Mus musculus OX=10090 GN=Ncdn PE=1 SV=1	AGDIDAKTR	0.521
45	Q8K0U4	Heat shock 70 kDa protein 12A OS=Mus musculus OX=10090 GN=Hspa12a PE=1 SV=1	FGLDPAVIKVR	0.803
46	Q8VEM8	Phosphate carrier protein, mitochondrial OS=Mus musculus OX=10090 GN=Slc25a3 PE=1 SV=1	IQTQPGYANTLR	0.709
47	Q8BGY7	Protein FAM210A OS=Mus musculus OX=10090 GN=Fam210a PE=1 SV=1	TSQETPSEKKEETDPLQDKSISLYQR	0.574
48	Q3UGY8	Brefeldin A-inhibited guanine nucleotide-exchange protein 3 OS=Mus musculus OX=10090 GN=Arfgef3 PE=1 SV=1	GAEGIKEQNQKER	0.658
49	Q8BPN8	DmX-like protein 2 OS=Mus musculus OX=10090 GN=Dmxl2 PE=1 SV=3	LTSLPPLYR	0.598
50	Q8BWF0	Succinate-semialdehyde dehydrogenase, mitochondrial OS=Mus musculus OX=10090 GN=Aldh5a1 PE=1 SV=1	GGLHADLLR	0.637
51	Q61316	Heat shock 70 kDa protein 4 OS=Mus musculus OX=10090 GN=Hspa4 PE=1 SV=1	YTENEGKMIMQDKLEKER	0.585
51	Q61316	Heat shock 70 kDa protein 4 OS=Mus musculus OX=10090 GN=Hspa4 PE=1 SV=1	YTENEGKMIMQDKLEKER	0.585
52	P48320	Glutamate decarboxylase 2 OS=Mus musculus OX=10090 GN=Gad2 PE=1 SV=1	LYGDSGKPAEGGGSVTSR	0.875
53	P63101	14-3-3 protein zeta/delta OS=Mus musculus OX=10090 GN=Ywhaz PE=1 SV=1	SSIEQKTEGAEKKQQMAR	0.663
54	Q8CGK7	Guanine nucleotide-binding protein G(olf) subunit alpha OS=Mus musculus OX=10090 GN=Gnal PE=1 SV=1	GNSSKTAEDQGVDEKER	0.530
55	Q99PT1	Rho GDP-dissociation inhibitor 1 OS=Mus musculus OX=10090 GN=Arhgdia PE=1 SV=3	ELDKDDESLR	0.605
56	P16858	Glyceraldehyde-3-phosphate dehydrogenase OS=Mus musculus OX=10090 GN=Gapdh PE=1 SV=2	KTVDGPSGKLWR	0.764
57	Q9WV92	Band 4.1-like protein 3 OS=Mus musculus OX=10090 GN=Epb41l3 PE=1 SV=1	VTPEKKAEEER	0.661
58	P80314	T-complex protein 1 subunit beta OS=Mus musculus OX=10090 GN=Cct2 PE=1 SV=4	KAGADEER	0.545
59	Q9WUL7	ADP-ribosylation factor-like protein 3 OS=Mus musculus OX=10090 GN=Arl3 PE=1 SV=1	KSAPDQEVR	0.610
60	O55100	Synaptogyrin-1 OS=Mus musculus OX=10090 GN=Syngr1 PE=1 SV=2	DVYFPQISSVKDR	0.517
61	P14873	Microtubule-associated protein 1B OS=Mus musculus OX=10090 GN=Map1b PE=1 SV=2	ASGPVKELEAER	0.552
62	P11031	Activated RNA polymerase II transcriptional coactivator p15 OS=Mus musculus OX=10090 GN=Sub1 PE=1 SV=3	LVSSSSSGSDSDSEVEKKLKR	1.544
63	P14231	Sodium/potassium-transporting ATPase subunit beta-2 OS=Mus musculus OX=10090 GN=Atp1b2 PE=1 SV=2	TVSDHTPKYQDR	0.515
64	Q99PT1	Rho GDP-dissociation inhibitor 1 OS=Mus musculus OX=10090 GN=Arhgdia PE=1 SV=3	LDKDDESLR	0.558
65	Q810U3	Neurofascin OS=Mus musculus OX=10090 GN=Nfasc PE=1 SV=1	LAALPKGRPDRPR	0.567
66	P11031	Activated RNA polymerase II transcriptional coactivator p15 OS=Mus musculus OX=10090 GN=Sub1 PE=1 SV=3	LASSKQSSSSR	2.292
67	Q9DBS2	Tumor protein p63-regulated gene 1-like protein OS=Mus musculus OX=10090 GN=Tprg1l PE=1 SV=1	VDSAGTSPTAVLAAGEDAGAGRPGAGTPLR	0.676
68	Q8K1M6	Dynamin-1-like protein OS=Mus musculus OX=10090 GN=Dnm1l PE=1 SV=2	ATSEALKISR	0.812
69	P14094	Sodium/potassium-transporting ATPase subunit beta-1 OS=Mus musculus OX=10090 GN=Atp1b1 PE=1 SV=1	ISELKPTYQDR	0.509
70	Q61183	Poly(A) polymerase alpha OS=Mus musculus OX=10090 GN=Papola PE=1 SV=4	PFPVTTQGSQQTQPPQR	0.838
71	Q9ERB5	Solute carrier organic anion transporter family member 1C1 OS=Mus musculus OX=10090 GN=Slco1c1 PE=1 SV=1	MDTSSKENAHLFHKN	0.733
72	P63038	60 kDa heat shock protein, mitochondrial OS=Mus musculus OX=10090 GN=Hspd1 PE=1 SV=1	TNEEAGDGTTTATVLAR	0.501
73	Q9JHU4	Cytoplasmic dynein 1 heavy chain 1 OS=Mus musculus OX=10090 GN=Dync1h1 PE=1 SV=2	EINPKVPVNLLR	0.550
74	Q9D289	Trafficking protein particle complex subunit 6B OS=Mus musculus OX=10090 GN=Trappc6b PE=1 SV=1	KSAEQGEVENGR	0.515
75	Q9D0F9	Phosphoglucomutase-1 OS=Mus musculus OX=10090 GN=Pgm1 PE=1 SV=4	VTVKTQAYPDQKPGTSGLR	0.740
76	P00848	ATP synthase subunit a OS=Mus musculus OX=10090 GN=Mtatp6 PE=1 SV=1	PSILFPSSKR	0.976
77	P39053	Dynamin-1 OS=Mus musculus OX=10090 GN=Dnm1 PE=1 SV=2	ANSDALKIAKEVDPQGQR	0.667
78	Q7TQD2	Tubulin polymerization-promoting protein OS=Mus musculus OX=10090 GN=Tppp PE=1 SV=1	TAAPELSALEEAFR	0.524
79	P56480	ATP synthase subunit beta, mitochondrial OS=Mus musculus OX=10090 GN=Atp5f1b PE=1 SV=2	KDATSKVALVYGQMNEPPGAR	0.556
80	Q8VED9	Galectin-related protein OS=Mus musculus OX=10090 GN=Lgalsl PE=1 SV=1	SPVQADVYFPR	0.580
81	P43006	Excitatory amino acid transporter 2 OS=Mus musculus OX=10090 GN=Slc1a2 PE=1 SV=1	EDNLGIDKR	0.521
82	Q9D2G2	Dihydrolipoyllysine-residue succinyltransferase component of 2-oxoglutarate dehydrogenase complex, mitochondrial OS=Mus m	EAGAAKGLR	0.529
83	Q62261	Spectrin beta chain, non-erythrocytic 1 OS=Mus musculus OX=10090 GN=Sptbn1 PE=1 SV=2	AESPDVKGR	0.547
84	Q8VEM8	Phosphate carrier protein, mitochondrial OS=Mus musculus OX=10090 GN=Slc25a3 PE=1 SV=1	TLKEDGVR	0.912
85	Q9JKC6	Cell cycle exit and neuronal differentiation protein 1 OS=Mus musculus OX=10090 GN=Cend1 PE=1 SV=1	ATSEPKGPGDGAEEDESNTGGR	0.555
86	Q80XU3	Nuclear ubiquitous casein and cyclin-dependent kinase substrate 1 OS=Mus musculus OX=10090 GN=Nucks1 PE=1 SV=1	SEDEKDDHKNVR	0.572
87	Q9JHK4	Geranylgeranyl transferase type-2 subunit alpha OS=Mus musculus OX=10090 GN=Rabggta PE=1 SV=1	TSEEQAEAKR	0.572
88	O88342	WD repeat-containing protein 1 OS=Mus musculus OX=10090 GN=Wdr1 PE=1 SV=3	SGKGHTNQVSR	0.854
89	Q9CPY7	Cytosol aminopeptidase OS=Mus musculus OX=10090 GN=Lap3 PE=1 SV=3	IQVDNTDAEGR	0.529
90	P09411	Phosphoglycerate kinase 1 OS=Mus musculus OX=10090 GN=Pgk1 PE=1 SV=4	AEPAKIDAFR	0.581
91	Q68FD5	Clathrin heavy chain 1 OS=Mus musculus OX=10090 GN=Cltc PE=1 SV=3	LLDALKNNRPSEGPLQTR	0.513
92	Q9CYT6	Adenylyl cyclase-associated protein 2 OS=Mus musculus OX=10090 GN=Cap2 PE=1 SV=1	EDKKEEPSPSR	0.509
93	Q01853	Transitional endoplasmic reticulum ATPase OS=Mus musculus OX=10090 GN=Vcp PE=1 SV=4	IDELDAIAPKR	0.602
94	Q99LX0	Parkinson disease protein 7 homolog OS=Mus musculus OX=10090 GN=Park7 PE=1 SV=1	GSHYSYSESR	0.975
95	Q6ZQ93	Ubiquitin carboxyl-terminal hydrolase 34 OS=Mus musculus OX=10090 GN=Usp34 PE=1 SV=3	KHVQQR	0.568
96	Q68FD5	Clathrin heavy chain 1 OS=Mus musculus OX=10090 GN=Cltc PE=1 SV=3	TAIKADR	0.506
97	Q9D0E1	Heterogeneous nuclear ribonucleoprotein M OS=Mus musculus OX=10090 GN=Hnrnpm PE=1 SV=3	GAPGPKGEERPTQNEKR	0.510
98	Q3UHD9	Arf-GAP with GTPase, ANK repeat and PH domain-containing protein 2 OS=Mus musculus OX=10090 GN=Agap2 PE=1 SV=1	VTTSGAKAGGGKGAGSR	0.678
99	Q2PFD7	PH and SEC7 domain-containing protein 3 OS=Mus musculus OX=10090 GN=Psd3 PE=1 SV=2	GTHPKTISR	0.623
100	Q68FD5	Clathrin heavy chain 1 OS=Mus musculus OX=10090 GN=Cltc PE=1 SV=3	FITEEDYQALR	0.644

B

1	Brevican		Schnittstelle	
2	Q61361	Brevican core protein OS=Mus musculus OX=10090 GN=Bcan PE=1 SV=2	RELE:TPSE	2.516
3	Q61361	Brevican core protein OS=Mus musculus OX=10090 GN=Bcan PE=1 SV=2	EALE:EEKE	2.069
4	Q61361	Brevican core protein OS=Mus musculus OX=10090 GN=Bcan PE=1 SV=2	EEEK:EQED	1.134
5				

Suppl. Figure 4. Observed overrepresented peptides from N-terminomics results. (A) Significantly overrepresented peptides summarised in a list. **(B)** An excerpt from this list focussing on Brevican and its cleavage sites.

Mean velocity does not change between tested groups in MWM test

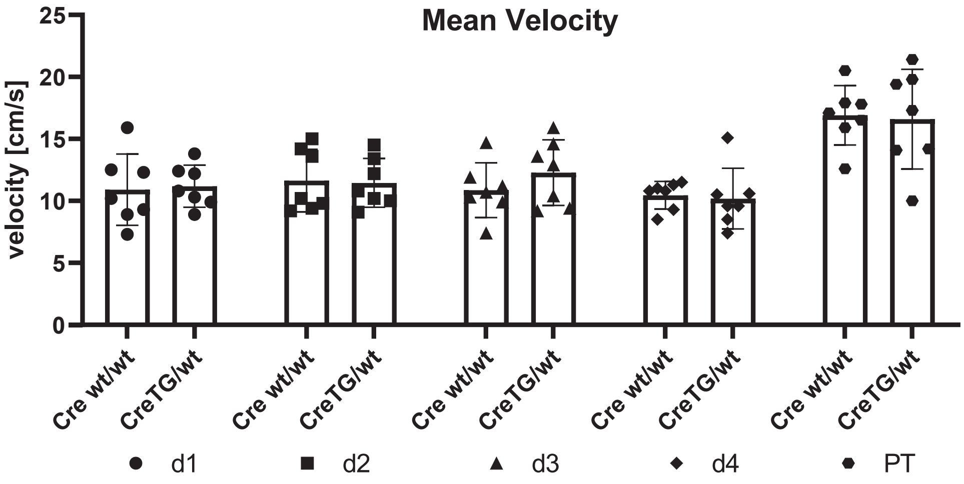

Suppl. Figure 5. Mean velocity does not change between tested groups in MWM test – During the training phase, mice swam for 90 seconds per session, while during the probe trial, they swam for 60 seconds. Analysis of swimming speed revealed no significant differences between the wt and transgenic groups across all testing days in the Morris Water Maze paradigm test. These findings indicate that the animals exhibit no locomotor impairments, suggesting that observed differences in performance are unrelated to motor function.

CRISPR-Cas9-mediated KO of meprin β in HEK293T cells

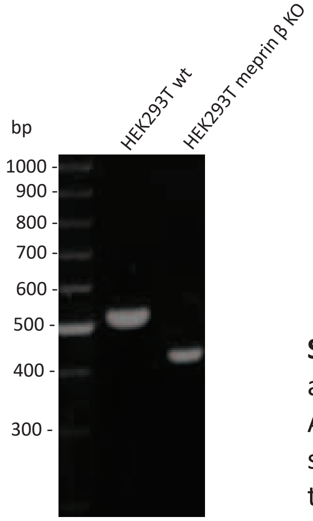

Suppl. Fig 6. Confirmation of KO. Genotype PCR of HEK293T and CRISPR/Cas9-generated HEK293T meprin β KO cells. Agarose gel was used to visualize meprin β-specific signals. The size shift of the PCR product validates a deletion in MEP1B in the HEK293T meprin β KO cells, leading to the knock-out.

Biotinylation of Surface Proteins from Primary Hippocampal Neurons

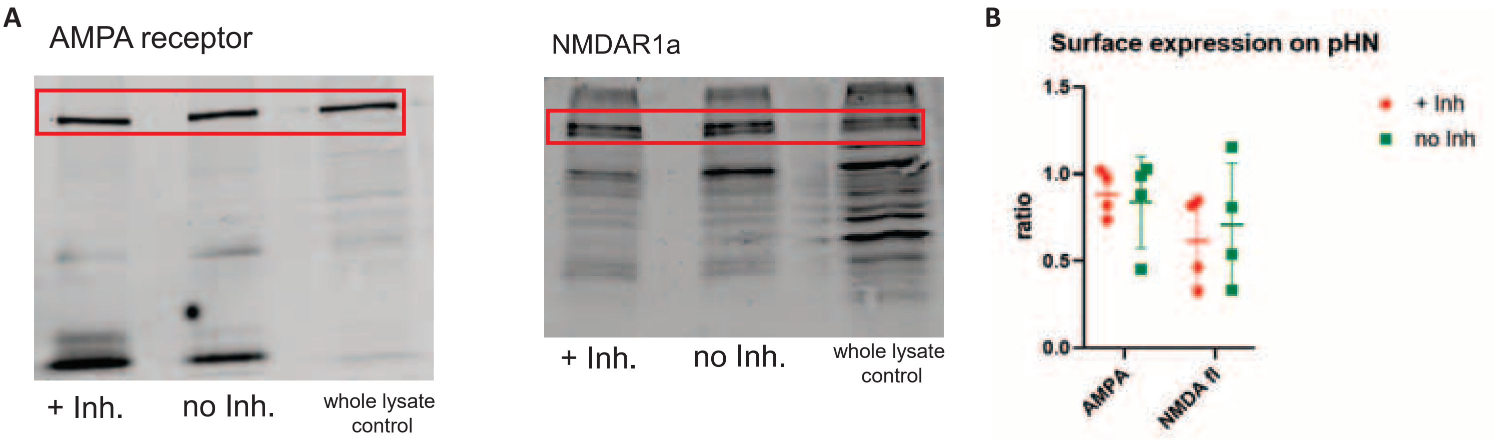

*1 of 4 representative experiments

Suppl. Figure 7. Biotinylation assay. Primary hippocampal neurons were extracted from P1 mice. For 72 hours we inhibited the cells with meprin β inhibitor actinonin prior to the biotinylation assay. The representative western blots reveal no differences of neither AMPA- nor NMDA-receptor expression (red brackets) on the surface of primary hippocampal neurons (A), which is also observed over all four expriments (B).

Hippocampal Paired-Pulse Ratio is unaffected by meprin β overexpression

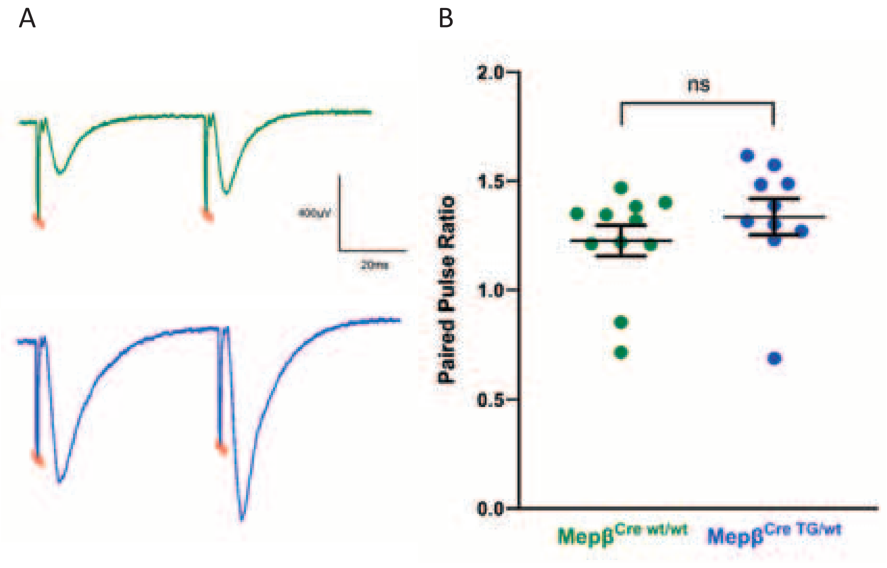

Suppl. Figure 8. Hippocampal Paired-Pulse Ratio is unaffected by meprin β overexpression. A. Representative evoked fEPSPs from a paired-pulse stimulus in wildtype mepβ$^{Cre;wt/wt}$ (green) and mepβ$^{Cre;TG/wt}$ (blue) with a 50ms interstimulus interval (ISI). **B.** Analysis (unpaired t-test) of the ratio of the second evoked fEPSP to the first evoked fEPSP showing no difference between genotypes.
